# Transition-Metal Clusters and Complexes: Ab Initio Studies on Inducing Photoexcitation and Photocatalytic Activity

**DOI:** 10.3390/ma19194047

**Published:** 2026-09-22

**Authors:** Ellie L. Uzunоva, Ivelina M. Georgieva, Tsvetan Zahariev

**Affiliations:** Institute of General and Inorganic Chemistry, Bulgarian Academy of Sciences, Acad, G. Bonchev Str., Block 11, 1113 Sofia, Bulgaria; ivelina@svr.igic.bas.bg (I.M.G.); tzahariev@svr.igic.bas.bg (T.Z.)

**Keywords:** density functional theory (DFT), chalcogenide complexes, water splitting, CO_2_ reduction, lanthanides, antenna chromophores

## Abstract

Theoretical methods are indispensable in studying photocatalytic reaction pathways, the energy barriers of each elementary reaction step and in elucidating the active sites in catalysis. Transition metal sulfides of the 3d elements, M = Fe, Co, Ni, Cu, mimic natural reductase and carbon monoxide dehydrogenase enzymes. The exact environment of enzyme centers, which includes a protein surrounding the active site and coordinated ligands, cannot be achieved by synthesis. Careful modeling of ligands coordinated to the active site can predict whether photoactivation occurs and ways to enhance visible light absorption. For heavier transition metals, such as the f-block elements, the photophysical properties become more pronounced. From the computational results, supported by experiments, sulfur emerges as a key element in promoting photochemical and photophysical activity. Bridging sulfur in the clusters and complexes of the 3d elements (M2-S2) acts as an electron donor similar to coordinated carbonyl or nitrosyl ligands. Lanthanides also take advantage of the coordination of sulfur-containing ligands to fine-tune light absorption and light emission, producing lanthanide sulfide photocatalysts or high-intensity luminescent complexes. The excited-state energy transfer mechanism and factors responsible for the specific lanthanide emission are successfully predicted by combined DFT/TDDFT, multireference and semiempirical calculations in close correlation with the experiment.

## 1. Electronic Structure Studies of Clusters and Complexes Which Mimic Natural Dehydrogenase and Carbon Monoxide Dehydrogenase Enzymes

The transition metal elements encompass two major assemblies: (i) elements with partly filled d-orbitals and (ii) elements with partly filled f-orbitals. The first transition row includes the elements with a partly filled 3d shell. Many of their compounds have intense light absorption bands. The TiO_2_ as electrode material was the first one studied for photochemical water splitting in the pioneering work of Fujishima in 1972 [1]. In the years to follow, a huge number of solids and molecular complexes of the 3d elements were studied in either water splitting or CO_2_ reduction [2,3,4,5,6,7,8,9,10,11,12,13,14,15]. Theoretical modeling went parallel with experimental studies and in this way reaction mechanisms were discovered, active sites were elucidated, and the road to new active photocatalytic compositions was traced [5,6,15,16,17,18,19,20,21]. Key parameters can be established by electronic structure methods: thermodynamic stability, proton and electron affinities, light absorption spectra [16,17,18,19,20,21]. Starting with TiO_2_, the studies continued with other oxide clusters and solids. In a solid crystalline material, the photochemical activity depends on the size of the optical band gap. In molecular systems the equivalent is the HOMO-LUMO gap, but in transition metal complexes there are often low-lying excitation states and their most intense excitation bands are determined by evaluation of the UV–vis spectra. TiO_2_, for example, and many other oxide materials have band gaps of the order or exceeding 3 eV and therefore require UV light for photoactivation [1,5,7,17,22]. The oxides of the late 3d elements (Fe, Co, Ni, Cu) feature smaller band gaps but still above 2 eV [22,23,24]. The cupric oxide (Cu_2_O) is among the promising materials for catalytic and photocatalytic water splitting [25,26], and nanoparticles are of particular interest in CO_2_ reduction [20]. The best option, however, is to create materials which absorb largely visible light, and the natural enzymes (ferredoxins, CODH—carbon monoxide dehydrogenase) became a prototype in creating synthetic analogs [8,9,10,11,15,27,28,29,30]. This trend became fruitful, as it introduced transition metal sulfides in the form of clusters, solids, and molecular complexes with variable ligands [8,9,10,11,12,13,14,15,18,19,20,21]. The sulfide materials have smaller band gaps than the oxides, and the light absorption bands in sulfide clusters and complexes can be modified by the coordination of ligands. The family of [Fe-Fe] iron hydrogenase enzymes and the complexes mimicking the enzyme active state were most extensively studied. The Fe_2_S_2_ rhombic core, part of redox enzyme structures, forms butterfly-like complexes with either nitrosyl or carbonyl ligands. The hexacarbonyl Fe_2_S_2_(CO)_6_ has light absorption bands in the visible region, manifested by a strong band at 460 nm [31]. Theoretical studies proved that all of the late 3d transition metals, Fe-Cu, form rhombic disulfides and coordinate ligands [18,21,32].

The electronic structure of the clusters and complexes can be best examined by density functional theory using hybrid functionals such as B3LYP, which was applied throughout our studies of carbonyl complexes, with results summarized in Table 1, Table 2, Table 3 and Table 4. The bare clusters were also studied by coupled-cluster methods and the excellent agreement between CCSD(T)- and B3LYP-calculated energies proved the reliability of the DFT methods for these clusters and complexes [18]. Water solvent was introduced via dielectric constant throughout reaction path following and structure optimization. These calculations provide accurate description of the frontier orbitals, the charge distribution, the proton and electron affinities, and the UV–vis spectra. Both the bare M_2_S_2_ clusters (M = Fe, Co, Ni, Cu) and their coordination compounds have a flexible geometry, and the M_2_S_2_ core can be either planar or deviate significantly from planarity. The global energy minimum structures of all M_2_S_2_(CO)_4_ complexes (M = Fe, Co, Ni, Cu) have a rhombic non-planar core M_2_S_2_. The energy gap between non-planar (global minima) and planar configurations is 67 kJ mol^−1^ for Fe_2_S_2_(CO)_4_ and 88 kJ mol^−1^ for Co_2_S_2_(CO)_4_ (Figure 1a,b). Up to six carbonyl ligands can be coordinated by M_2_S_2_, three on each metal center, to form a hexacarbonyl (Figure 1c). With nitrosyl ligands, the tetra-nitrosyl was the highest coordination reported (Figure 1d).

**Table 1:** Dissociation energies (D_zpe_) for the loss of carbonyl ligands of [M2-X2] hexa- and tetracarbonyls, M = Fe or Co; X = S or Se, calculated by DFT (B3LYP) in water as solvent, corrected for zero-point energy. Data apply to global minima configurations.

Complexes	D_zpe_ kJ mol^−1^
Fe_2_S_2_(CO)_6_ → Fe_2_S_2_(CO)_5_ + CO	154
[Fe_2_(S_2_H)](CO)_6_ ⇒ [Fe_2_(S_2_H)](CO)_5_ + CO	163
Co_2_S_2_(CO)_6_ → Co_2_S_2_(CO)_5_ + CO	150
[Co_2_(S_2_H)](CO)_6_ ⇒ [Co_2_(S_2_H)](CO)_5_ + CO	151
Co_2_Se_2_(CO)_6_ → Co_2_Se_2_(CO)_5_ + CO	12
Fe_2_Se_2_(CO)_6_ → Fe_2_Se_2_(CO)_5_ + CO	18
Co_2_Se_2_(CO)_4_ → Co_2_Se_2_(CO)_3_ + CO	197
Fe_2_Se_2_(CO)_4_ → Fe_2_Se_2_(CO)_3_ + CO	204

**Table 2:** Calculated proton affinities (PA, kJ mol^−1^) at sulfur centers and [H^+^,e^−^] affinities at either sulfur or at Co or Fe centers (kJ mol^−1^) for [M2-S2] and [M2-Se2] carbonyl complexes. n = 0 for [H^+^,e^−^] attachment, n = 1 for protonation.

	PA	[H^+^,e^−^]	2[H^+^,e^−^]
[Co_2_(S_2_)H]^n+^(CO)_6_; S-H	851	252	474
[Co_2_H(S_2_)](CO)_6_; Co-H		173	
[Co_2_S_2_H]^n+^(CO)_4_; S-H	767	224	
[Co_2_HS_2_](CO)_4_; Co-H-Co		177	
[Fe_2_(S_2_)H]^n+^(CO)_6_; S-H	717	235	445
[Fe_2_H(S_2_)](CO)_6_; Fe-H		212	
[Fe_2_S_2_H]^+^(CO)_4_; S-H	651		
[Fe_2_HS_2_](CO)_4_; Fe-H-Fe		229	
[Fe_2_Se_2_H]^+^(CO)_4_; Se-H	639		
[Fe_2_HSe_2_](CO)_4_; Fe-H-Fe		228	
[Co_2_Se_2_H]^n+^(CO)_4_; Se-H	748	191	
[Co_2_HSe_2_](CO)_4_; Co-H-Co		179	
[Co_2_HSe_2_](CO)_4_; Co-H		201	
[Ni_2_S_2_H]^n+^(CO)_4_; S-H	935	181	
[Ni_2_Se_2_H]^n+^(CO)_4_; Se-H	907	168	
[Cu_2_S_2_H]^n+^(CO)_4_; S-H	968	134	
[Cu_2_Se_2_H]^n+^(CO)_4_; Se-H	936	81	

**Table 3:** TD-DFT results for selected complexes of reduced (H+,e^−^) cobalt sulfides and selenides. The most intense light absorption bands are listed. The results are from refs. [18,21,33].

Complex	Light Absorption Bands, nm	Oscillator Strength
[Co_2_S-SH](CO)_6_	643; 873	0.0039; 0.0061
[Co_2_S-SH](CO)_4_	689; 824	0.0060; 0.0010
[Co-H-CoS_2_](CO)_4_	750; 803	0.0020; 0.0040
[Fe_2_S-SH](CO)_6_	727; 874; 1021	0.0020; 0.0040; 0.0144
[Co_2_Se-SeH](CO)_4_	628; 749	0.0011; 0.0017
[Co-H-CoSe_2_](CO)_4_	748; 1610	0.0006; 0.0250
[Co(Co-H)Se_2_](CO)_4_	546; 639	0.0017; 0.0024

**Table 4:** TD-DFT results for OER intermediates of selected sulfide and selenide tetracarbonyl complexes. The most intense light absorption bands are listed. The results are from ref. [21].

Complex	Light Absorption Bands, nm	Oscillator Strength
Co_2_S_2_(CO)_4_; O-O	651	0.0014
Co_2_Se_2_(CO)_4_; O-O	582	0.0091
Fe_2_Se_2_(CO)_4_; O-O	807	0.0131
Cu_2_Se_2_(CO)_4_; O-O	430	0.0094
Co_2_S_2_(CO)_4_; OOH^•^	507; 573	0.0023; 0.0027
Co_2_Se_2_(CO)_4_; OOH^•^	531	0.0014
Fe_2_Se_2_(CO)_4_; OOH^•^	798	0.0052
Cu_2_Se_2_(CO)_4_; OOH^•^	590	0.0130
Ni_2_Se_2_(CO)_4_; OOH^•^	558	0.0023

The flexibility of the M_2_S_2_ core is a result of the highly delocalized bonding orbitals in these compounds (Figure 2). The electronegativity of sulfur is much lower than that of oxygen, and the M-S bonds have lower polarity than the M-S bonds. As a result, the frontier orbitals and the overall electron density are more evenly distributed in metal sulfides than in metal oxides [18]. This impacts the readiness of sulfides to accept and release electrons and protons, and proton–electron couples, respectively [18,19]. In the global minimum configuration of Fe_2_S_2_(CO)_6_, the HOMO features Fe-Fe and S-S binding and is delocalized over the Fe_2_S_2_ core and the carbon atoms from the carbonyl group, while the LUMO is largely a non-bonding orbital located on sulfur centers. The HOMO-LUMO gap is relatively large, 3.92 eV. The flat-core isomer has its HOMO as non-bonding orbitals on sulfur atoms; the LUMO features higher delocalization. Here, the HOMO-LUMO gap becomes much smaller, 2.49 eV. As the two isomers are separated by an energy gap of 0.923 eV, and both are diamagnetic, light absorption can easily induce isomerization. This structure flexibility plays a major role in the ability to efficiently absorb reactants and allows monitoring of photoactivation.

The reactions of water splitting and of CO_2_ reduction to either carbon monoxide (CO), formic acid (HCOOH), methanol (CH_3_OH) or even deeper reduction to hydrocarbons are all highly demanding in energy. Just the one-electron reduction of CO_2_ to form the radical-anion CO_2_^•−^ is endothermic and requires –206.5 kJ mol^−1^. The situation changes when protons are included, in a mechanism following the route of CODH (carbon monoxide dehydrogenase) enzymes: CO_2_ + 2H^+^ +2e^−^ ⇒ CO + H_2_O already needs 74 kJ mol^−1^, and the more desirable reaction to formic acid CO_2_ + 2H^+^ +2e^−^ ⇒ HCOOH needs slightly higher energy of 82 kJ mol^−1^ [9]. Numerous studies proved that the enzyme-mimicking transition-metal disulfides catalyze both water splitting and carbon dioxide reduction [18,19,20,21,29,33]. It has been argued that sulfide-based catalysts may be unstable and undergo transformation to hydroxides or oxo-hydroxides in course of the reaction of water splitting [14]. This, however, depends on the catalyst design. The thermodynamic stability of the disulfides as cores in coordination compounds was proven to be sufficiently high [8,9,11,15,18,19,21,29]. In water as solvent, iron sulfide clusters are stabilized by the coordination of water molecules [34,35]. Ni-Fe sulfide photocatalysts, the direct analogues of CODH enzymes, also proved stable to fragmentation in thermodynamical studies [15]. The fragmentation reactions of hexacarbonyl complexes may start with the loss of a carbonyl ligand [18,31,33], and this is crucial for the selenide complexes [21]. An example of the loss of carbonyl ligand from tetracarbonyl and hexacarbonyl complexes of cobalt and iron chalcogenides M2-X2 is presented in Table 1 [18,21,33]. In Fe_2_S_2_(CO)_6_ and Co_2_S_2_(CO)_6_, the loss of a carbonyl ligand from the hydrogenated complexes (S-H bond) is higher than from the parent complexes [18,33]. The loss of carbonyl ligand from a hexacarbonyl with selenide cluster core M2-Se2, M = Co, Fe is highly favorable, requiring energies of 12–18 kJ mol^−1^. The loss of a carbonyl ligand from a tetracarbonyl, however, requires 197–204 kJ mol^−1^; thus, selenides are not stable as hexacarbonyls, but as tetracarbonyls [21]. The coordination of a hydride ligand at sulfur leads to minor stabilization of the complexes and slightly higher binding energies of the carbonyl ligands. It is worth noting that the complexes are stable towards hydrolysis as well [33].

As proton transfers play a major role in CO_2_ reduction, the proton affinity and the proton–electron affinity of the catalysts are key markers in predicting their activity. Moreover, it has been experimentally determined, and proven in computational studies, that transition metal disulfide complexes with an M_2_S_2_ core attach hydrogen as a ligand. While protonation always occurs at a sulfur center, hydrogen is attached either at the sulfur or at the metal cation center, usually midway coordinated to both cations (Figure 3). The highest energy gap of 79 kJ mol^−1^ between the S-H bonded hydrogen and the M-H-M bonded hydrogen is found for the cobalt–sulfur hexacarbonyl complex Co_2_S_2_(CO)_6_ (Table 2). In the tetracarbonyl complex, however, this gap is reduced to 47 kJ mol^−1^ and the interconversion between the two forms is much easier to achieve, as shown in Figure 3. These results illustrate the possibility of finely tuning the properties of these complexes by simply changing the number of ligands. For the Fe_2_S_2_(CO)_6_ complex, the energy difference between the two isomeric hydrogen-bonded complexes is even smaller, 23 kJ mol^−1^, which indicates that the two forms can coexist.

The proton affinity is always much higher than the proton–electron affinity (Table 2). As protons are always attached at sulfur, the proton–electron couples will also form on sulfur, and the activation of the complexes to the active state of easily transferrable hydrogen can be reached by visible light irradiation [18,21,33]. Similar trends in ligand binding, proton affinities and proton–electron affinities are observed with the next element of the chalcogenide family, selenium, and the selenide complexes with an [M2-Se2] core [21]. Chalcogenide materials and, more specifically, selenides, have been explored in electro-catalysis for HER (hydrogen evolution reaction), OER (oxygen evolution reaction) and also as energy storage materials [13,36,37]. As structural analogs of the rhombic transition metal disulfides, they differ by the longer M-Se bonds, the preference for a non-planar structure, and higher stability of tetracarbonyl complexes over hexacarbonyl complexes [21]. The proton affinities of selenide complexes are lower than those of sulfide complexes, but the proton–electron affinities are quite similar.

## 2. Light Absorption Properties of Chalcogenide Complexes of the 3d Elements and Photocatalytic Activity in Water Splitting

After the first elementary step of proton–electron couple [H^+^,e^−^] attachment, all of the complexes in their reduced form absorb light in the visible and near-IR range (Table 3). Some complexes, namely [Fe_2_S-SH](CO)_6_ and [Co-H-CoSe_2_](CO)_4_, have very intense lines in the near-IR region. Thus, with respect to photoactivation in the HER reaction or in CO_2_ reduction, all of the selenide and sulfide complexes with carbonyl ligands of the late 3d elements (Fe, Co, Ni, Cu) are eligible. It has been proven experimentally that Fe_2_S_2_(CO)_6_ efficiently splits water upon visible light irradiation and, moreover, it is able to operate as a catalyst in dark conditions [11]. Though hexacarbonyl complexes are stable in solution and as bulk materials, visible or UV light irradiation triggers ligand loss and even M-S bond breakup [31,38]. After the first carbonyl ligand loss, however, the dissociation energy for the next carbonyl ligand loss increases, as shown in Table 1, and tetracarbonyl complexes are as efficient redox catalysts as the hexacarbonyl ones. Moreover, the carbonyl complexes with an M_2_S_2_ cluster core (M = Fe, Co, Ni, Cu) do not operate as bulk catalysts; they are carefully layered with InP (indium phosphide) to form an electrode material, which enhances their overall stability [11]. Theoretical modeling of the HER on selenide and sulfide complexes revealed that the iron sulfide and iron selenide complexes are most efficient [18,21] (Figure 4).

The reaction bottleneck, however, is the oxygen evolution reaction, OER [5,6,18,21]. The activation barriers are much higher than those for HER. The reaction pathway is pH-dependent because different intermediate species are formed in alkaline or acid media: peroxo- or hydroperoxo- species, (M_2_-O-O) or (M_2_-OOH). The route via an (M_2_-OOH) intermediate is more favorable, and the selenide and sulfide complexes of cobalt and iron are more efficient than those of nickel and copper (Figure 4). For the OER, photoactivation is of higher importance than it is for HER because of the higher energy barriers, particularly in the lack of acidity. Fortunately, all of the chalcogenide complexes with oxygen intermediates have their most intense light absorption bands in the visible region (Table 4).

## 3. Photocatalytic Activity of M_2_S_2_(CO)_6_ Complexes (M = Co, Fe) in Carbon Dioxide and Nitrogen Oxide Reduction

The reaction mechanisms of energy demanding redox processes such as CO_2_ and NO reduction, catalyzed by the hexacarbonyl complexes with Fe_2_S_2_ and Co_2_S_2_ rhombic cores, were studied by transition state theory in combination with density functional methods. Nitrogen oxide reduction efficiently proceeds on Fe_2_S_2_(CO)_6_ and Co_2_S_2_(CO)_6_, the latter complex being more efficient, by accepting an electron–proton couple to the dimeric form (NO)_2_. The N_2_O_2_H^•^ formed in the first reaction step is subsequently reduced to N_2_O and H_2_O [32]. The energy barriers to N_2_O formation are within 33–68 kJ mol^−1^ and, while these reaction steps would benefit from photoactivation, the next stage, N_2_O reduction, is more challenging, as the barriers range from 77 to 133 kJ mol^−1^. The first elementary step of all redox processes, in which M_2_S_2_(CO)_n_ (n = 4, 6) complexes are involved, include the attachment of the proton–electron couple, represented as a hydrogen ligand (1). It is subsequently transferred to water molecules, (NO)_2_ or CO_2_ reactants [32,33].

(1) [M_2_S_2_](CO)_6_ + [H^+^,e^−^] ⇒ [M_2_S_2_H](CO)_6_ + hν ⇒ [MHMS_2_](CO)_6_, where M = Co, Fe

Two different intermediates are formed in the first reduction step of CO_2_: (i) hydrogen attaches to the carbon atom to form an OCHO^•^ intermediate, and (ii) hydrogen attaches to the oxygen end of CO_2_ to form COOH^•^. The OCHO^•^ intermediate reacts further through hydrogenation of the oxygen end and formation of formic acid HCOOH as a final product. The COOH^•^ intermediate also reacts further trough hydrogenation of the other oxygen end-atom and thus leads to CO and H_2_O as final products. A selective catalyst can predetermine the reaction products of CO_2_ two-proton-two-electron reduction. The Fe_2_S_2_(CO)_6_ complex favors the formation of a COOH^•^ intermediate with an energy barrier of 87 kJ mol^−1^ and thus CO and water end products [33]. Though HCOOH can also be formed from the COOH^•^ intermediate by a second hydrogenation at the carbon center, this step is less favorable energetically. The Co_2_S_2_(CO)_6_ complex favors the formation of an OCHO^•^ intermediate with an energy barrier of 53 kJ mol^−1^, which yields selectively HCOOH as the end product. With respect to CO_2_ reduction, the Fe_2_S_2_(CO)_6_ complex is less selective than Co_2_S_2_(CO)_6_. Moreover, Fe_2_S_2_(CO)_6_, as a structural analog of hydrogenase enzymes, has high activity in HER and it runs as a side reaction.

Among the 4d elements, (Y-Ag), notable redox and hydrogenation catalysts, are present, among which the molybdenum disulfide, MoS_2_, is an example of a catalyst with widespread application: cocatalyst in hydrodesulfurization, hydrogenation catalyst in organic chemistry; electronic and optical properties of the material in 2D layers. As either layered material, or as nanoclusters, MoS_2_ proved efficient in photochemical water splitting [39,40]. When turning to the 4f elements, considered as inner-transition metals, the light absorption and photochemical properties are mixed with light-emission properties, again tunable by the proper choice of ligands. Photochemical and photophysical properties go hand-in-hand in the lanthanides.

## 4. Sulfur-Containing Organic Chromophores Enabling Charge Transfer Processes in Ln^3+^ Complexes

The sensitization ability of the ligand chromophore is specific for the luminescence of certain Ln(III) and the antenna effect could be achieved by UV excitation of new antenna ligands or various combinations of known ligand chromophores [41,42,43,44]. Along with N,N- and O,O-donor ligands, the antenna potential of the S,S-donor ligand (dithiocarbamate (dtc) derivatives) in sensitizing lanthanide luminescence are of research interest from experimental and theoretical perspectives [45,46,47,48]. These compounds have been applied successfully in the production of lanthanide sulfide nanoparticles, for the purposes of photocatalysis sulfides [49,50] and as suitable materials for novel electronic and optical devices [45,51,52]. For the luminescent lanthanide complexes operating via the antenna effect, the incorporation of sulfur-chelate chromophores into the structure of Ln^3+^ complexes (in particular, dithiocarbamate anion) and the formation of anionic [Ln(S_2_CNR_2_)_4_]^−^ and neutral Ln(S_2_CNR_2_)_3_ complexes are considered promising strategies to improve the resonance energy transfer to the lanthanide ion. Compared to the O-donor ligands, the S-donor chromophores increase the HOMO energy, decrease the HOMO−LUMO gap and may lead to enhanced electronic communication between the sulfur donor ligand and the lanthanide center. The S-donor chromophores are some of the strongest reducing agents and are considered to contribute to the low-lying ligand-to-metal charge transfer (LMCT) state of the Ln complexes [53,54,55,56,57,58,59]. It is known that the N,N-donor chromophores such as *phen* (1,10-phenanthroline) and *bipy* (2,2′-bipyridine) enhance the luminescence of Eu^3+^ and Tb^3+^ in their complexes, but this does not occur for Sm^3+^, Dy^3+^ and Pr^3+^ complexes [60]. The combination of *phen* and *dtc* ligands, however, results in the sensitization of Sm^3+^ and Pr^3+^ luminescence and the corresponding complexes exhibit more intense emission than those of the Eu^3+^, Tb^3+^ and Dy^3+^ analogues [44,54,61]. Heteroleptic lanthanide complexes containing two chromophore ligands, one of which is a *dtc* derivative, are suitable objects for estimating the type and role of charge transfer states in sensitizing lanthanide luminescence, opening an interesting perspective for tuning the optical properties of the lanthanide complexes [48].

A few hypotheses related to the emergence of charge transfer (CT) states in the excitation spectra of europium complexes with *dtc^−^* have been proposed to understand the different impact of the *dtc* ligand on the optical properties of the Eu^3+^-*phen* and Sm^3+^-*phen* complexes, particularly the inexplicably weak red emission of Eu^3+^: low-energy ligand-to-metal charge transfer (LMCT) state [53], which is the most efficient quenching channel for Eu^3+^ luminescence [53,54,55,58] or interligand charge transfer (ILCT) character of the lowest excited state [62]. Although the LMCT states can contribute to the sensitization mechanism of the lanthanide complexes, the corresponding molar absorption coefficients are rather modest compared to the intraligand transitions. Another excited-state phenomenon, photoinduced electron transfer (PET), was also considered a typical quenching mechanism when the excited Ln^2+^/L^•^ state is lower in energy than that of Ln^3+^ [63,64]. The R_2_dtc^−^ is expected to be a redox-active ligand toward Eu^3+^ because of the low oxidation potential of the *dtc* anion (producing a radical (R_2_NCS_2_^−^/R_2_NCS_2_^•^, ~0.24V)) [65] and the most reducible Eu^3+^ (reduction potential Eu^3+^/Eu^2+^_aq_ = −0.34 V) [66]. The presence of *dtc* ligands in Eu^3+^-*phen* complexes, unlike in the Sm^3+^-*phen* complex, leads to changes in UV–vis absorption, especially in the visible range, and consequently to variations in the color of the complex, depending on the environment and temperature. In solution (dichloromethane), the complex is colored in yellow, whereas in solid state, it assumes an unusual red-orange color. Upon cooling at 77 K, the red-orange complex in solid state turns yellow. The luminescence intensity of the Eu^3+^ complex could be modulated by variation in the substituents in *dtc*; for example, it increases when the *pyrrolidine* substituent in the *dtc* ligand is replaced by a *diethyl* substituent. Combined experimental and theoretical approaches, including ab initio, DFT and semiempirical methods, were developed and applied for a thorough description of the *antenna* mechanism in luminescent lanthanide complexes from ligand-centered absorption (S_0_ → S_n_), geometry relaxation to the first singlet state, S_n_ → S_1_ (Kasha’s rule) and radiative relaxation S_1_ → S_0_ (fluorescence) or nonradiative intersystem crossing S_1_ → T_1_ to radiative (phosphorescence) and nonradiative T_1_ → S_0_ relaxation or ligand-to-metal energy transfer (*dtc* → Eu^3+^) in an excited state and metal-centered emission (luminescence from populated excited-state acceptor levels ^5^D_0_, ^5^D_1_ for Eu^3+^). The Ln(S_2_CR)_3_(phen) complexes studied, (Ln ≡ Eu, Sm, *phen* ≡ 1,10-phenanthroline and R ≡ N(C_2_H_5_)_2_, N(CH_2_)_4_) could be considered as benchmark models of the heteroligand luminescent lanthanide complexes with *dtc* [48]. The calculated energy level diagrams and rates of competitive radiation and nonradiation rates of the isostructural Eu^3+^ and Sm^3+^ complexes in the gas phase and solution revealed the following: (i) similar energy level diagrams; (ii) ILCT (*dtc*-to-*phen*) character of the S_1_ state and a small LMCT contribution to the S_1_ state only for Eu(Et_2_dtc)_3_(phen) complexes with Et_2_dtc, without effect on the S_1_ state energy; (iii) comparable electronic relaxation paths; and (iv) a suitable energy gap between ligand-centered donor and metal-centered acceptor states for efficient L-to-Ln energy transfer (Figure 5) [48]. No distinct low-lying LMCT state was identified within the computational study. Therefore, a dominant LMCT-mediated quenching pathway appears unlikely, although a minor LMCT contribution cannot be completely excluded.

Two pathways were predicted for Eu^3+^ and Sm^3+^ complexes in solution: S_0_ → S_n_ → S_1min_ → T_1_ → Ln^3+^ or S_0_ → S_n_ → S_1min_ → Ln^3+^ (Figure 5). The predicted similar relaxation paths and the appropriate D*-A* energy gap favor the luminescent ability for both complexes. The evaluation of the antenna mechanism alone does not reproduce the difference in the luminescence quantum yield (QY) of Eu^3+^ and Sm^3+^. In addition to the sensitized luminescence mechanism (antenna effect), the degree of charge transfer, electron density distribution in the ground state and the formation of radical species turn out to be very important factors, especially for S,S-ligands in the Ln(III) complexes. The calculated Ln–pdtc/phen bonding, charge decomposition analysis (CDA), Mayer bond order, and overlap populations predicted a larger L(dtc)-to-M(Eu^3+^) charge transfer compared to that in the Sm^3+^ complexes. An important conclusion can be drawn, namely, that redox-activity of the *pdtc* anion ligand mainly toward Eu^3+^ occurs in the ground electronic state. These results were supported by the EPR measurements, which provided important information on the appearance of signals for *pdtc* radical and Eu^2+^ in the ground state (without UV excitation). Further, the EPR measurements at room and low temperatures, with and without UV excitation, provide important information on the radical species formation and its dynamics under varying conditions. In the case of an increase in the amount of *dtc* radicals, the charge transfer character of excitation from ligand (*dtc*) to *phen* (ILCT) or to the metal is expected. The experimentally predicted charge transfer upon excitation was supported by the calculated S_1_ state character, namely *dtc*-to-*phen*, as well as a small *dtc*-to-Ln^3+^ charge transfer for Eu(Et_2_dtc)_3_(phen). In the Eu(dtc)_3_(phen) complexes, there is a special case in which two types of charge transfer are observed: (i) LMCT occurs in the ground state, producing a partial Eu^2+^ and *dtc* anion radical and (ii) photoinduced charge transfer proceeds in the S_1_ state, which has an interligand *dtc*-to-*phen* charge transfer character. Computations showed that the *dtc* ligand with partial radical character in the europium complex produces bands which contributed to the observed absorption of the europium complex in the 480–600 nm visible range and red-orange color in solid state. Another explanation for the intense long-wavelength absorption (the red-brown color of the solid-state Eu^3+^ complex at 458 nm) is a possible overlap between the ILCT state and Eu^2+^ 4f^7^ → 4f^6^ 5d^1^ transition (a Laporte-allowed transition at ∼400 ÷ 480 nm) to a large extent due to their close excitation energies. To a lesser extent, the partial interaction between the ILCT state and the Eu^3+^ electric dipole *f*-*f* states (460–470 nm, ^7^F_0_ → ^5^D_2_ and 470–480 nm, ^7^F_1_ → ^5^D_2_) is also expected. At a low temperature (77 K), this interaction breaks due to the blueshift of the ILCT band to ∼400–420 nm and as a result, the color of Eu(pdtc)_3_(phen) complex changes from red-orange to yellow. The increase in vertical excitation energy (blueshifted band) with decreasing temperature is explained by the large electron population of the lowest vibrational states of the ground electronic state. The combined approach of theoretical and experimental studies thoroughly reveals the factors (solvent, strength of intermolecular interactions in a saturated solution or the solid state, Ln^3+^ type and temperature) that affect the UV–vis spectra (position and intensity of absorption bands). Furthermore, they could be used to tune the absorption ability of lanthanide complexes in relation to the mechanism of sensitized luminescence. The relatively weak emission of the europium complex was explained by partial reduction of Eu^3+^ in the ground state.

## 5. Theoretical Description of Photophysical Processes in Ln^3+^ Complexes with Antenna Chromophores

Lanthanide luminescence is a unique workable property, but its intensity is weak because of the small molar absorption coefficients of 4f-4f transitions of lanthanide ions (Ln^3+^) (not higher than 10 M^−1^ cm^−1^). With the discovery of the possibility of enhancing the luminescence intensity of Ln^3+^ by introducing ligand chromophores into the coordination sphere of lanthanides, the complexes have attracted great attention as clean energy conversion materials utilizing ligands for energy absorption and efficient photoexcitation leading to lanthanide emission [67]. Lanthanide luminescence is characterized by well-defined narrow bands in various spectral ranges (including the visible region), relatively long lifetimes and high quantum yields. The luminescence quantum efficiency (η) and yield (QY) can be modulated by varying the Ln(III) and the chromophores, called *antenna* ligands, which transfer the absorbed UV energy to the 4f excited state of the lanthanide ion by a nonradiative mechanism. The optical characteristics of the luminescent lanthanide complexes have found numerous technological applications in organic light-emitting diodes (OLEDs), telecommunications, lasers, security marking, barcoding, luminescent probes in biomedical analysis, time-resolved microscopy, luminescent chemical sensors, coatings for photovoltaics, luminescent molecular thermometers, immunoassays or agriculture [68,69,70,71]. The basic steps of the energy transfer mechanism and the key factors responsible for efficient excited-state energy transfer are well known. The energy gap between the triplet donor state and the acceptor level of the specific Ln^3+^ (ΔE_L-Ln_) in the range 0.35–0.6 eV, according to the Latva’s empirical rule is considered an important requirement for energy transfer [72]. Numerous synthesized homo- and heteroleptic lanthanide complexes aim to fulfill the established conditions and the most studied antenna ligands, which have high thermal and chemical stability and good binding ability to the lanthanide ions (La^3+^, Pr^3+^, Sm^3+^, Eu^3+^, Gd^3+^, Tb^3+^, Dy^3+^) are N,N-donor heterocycles such as *phen* (1,10-phenanthroline) and *bipy* (2,2′-bipyridine) [73,74,75], O,O-diketones [76,77] and S,S-dtc [54,78]. Recently, a multi-functional, highly active, and versatile catalyst that self-assembles in situ from a light-harvesting polyamine ligand and a stable Sm(III) precursor for the capture and efficient visible-light-driven CO_2_ reduction has been reported. This catalyst generates either CO or HCO_2_H in a reaction-condition-dependent manner with >99% selectivity for both products and with the highest turnover number for CO generation reported to date for any photocatalytic system, including heterogeneous ones. This is the first Ln-based catalytic process for CO_2_ reduction, the first Ln(II)-catalyzed reduction of CO to MeOH and CH_4_, and the first Ln(II)-catalyzed H_2_ production [79].

Improved experimental spectroscopic techniques and theoretical approaches over the last decade have revealed a more complex mechanism of the energy transfers operating in the antenna effect encompassing all possible competing excited-state processes and selection rules as well as the luminescence sensitization via ligand singlet state and charge transfer states [48,57,80,81,82,83,84]. This section highlights the progress in theoretical approaches to simulate and elucidate the sensitization mechanism in luminescent lanthanide complexes by the *antenna* effect. The general scheme and the main known steps of the sensitization mechanism were first summarized by Andrade et al. [85]: (1) intense absorption of UV radiation by the sensitizing ligand chromophores; (2) rapid relaxation of the ligands to their lowest triplet state; (3) energy transfer from the triplet state to a quasi-resonant energy state of the lanthanide ion; (4) decay of the excited lanthanide metal ion to the ground state via photon emission in the visible region. Initially, the experimental UV–vis f-f excitations in the lanthanide ion were used to estimate the Judd–Ofelt intensity parameters Ω_λ_, which were further processed to derive the Einstein coefficients A for each of the ^5^D_0_ → ^7^F_J_ transitions. From these, branching ratios (relative intensities of each transition in the emission spectra), radiative decay rates and quantum yields were determined [86]. A number of structural, electronic and energetic parameters of lanthanide complexes as a function of the ligand type have been calculated and analyzed to predict a relation between the luminescence quantum yield and specific ground-state properties [87,88]. In a series of Ln^3+^ complexes with coumarin-3-carboxylic acid, a correlation between specific Ln-O bonding characteristics in the ground state, especially a larger orbital interaction term (covalent contributions) and shorter Ln-O bond lengths calculated with the DFT/B3LYP method were found to correlate with a larger luminescence quantum yield [89]. The efficiency of ligand-to-lanthanide energy transfer was assessed in the pioneering work of Freidzon et al. at the CASSCF level based on the relative energies of the triplet excited states with respect to the emitting levels of Ln^3+^ [90]. The luminescent properties of the lanthanide complexes were successfully modeled with a strategy that unites experimental techniques and theoretical approaches in close relation to each other [91] and the execution of the procedure was included in the LUMPAC program code, developed for Eu^3+^ complexes [92]. For large lanthanide systems (such as amorphous silicates, organic–inorganic hybrids and crystalline metal–organic frameworks containing lanthanide ions), the semiempirical Sparkle model [88] has been developed to determine ground-state geometries with accuracy similar to that of ab initio/ECP calculations and significantly lower CPU times [93,94]. To assess the rates of energy transfer between the ligand and Ln^3+^_,_ Malta and collaborators [95] developed a formalism based on the Judd–Ofelt intensity parameters that takes into account the singlet and triplet excited states of the complex [96]. For the evaluation of the latter, semiempirical methods (INDO/S-CIS) can be employed, providing good estimates of excitation energies and oscillator strengths. The described theoretical approaches, however, do not give a detailed description of the discrete processes and responsible factors that govern the photoluminescence.

The prospective design of new highly luminescent lanthanide complexes requires a thorough understanding of the relationship between the composition, geometry and photophysical properties and processes. An advanced entire computational strategy for reconstruction of the *antenna*-effect mechanism for lanthanide complexes has recently been developed [48,80,81,82,83,84] and relies on ab initio, semiempirical and the Judd–Ofelt and Malta methods. The computational approach is based on the long-established concept that the excited electronic states involved in the most intensive absorption transitions are localized on the antenna ligands, while the main emissive states are metal-centered and weakly perturbed by the ligand field present. It is then possible to apply certain approximations, in which a quantum-chemical modeling of the ligand-centered states can be performed, while the electronic structure of the lanthanide, including the 4*f* subshells is instead treated in the form of an effective potential. This assumption allows lanthanide complexes to be considered in a singlet ground state. The data for the relevant *f*-*f* absorption and emission transitions are normally extracted from experimental measurements or evaluated empirically, using data reported in available sources, such as the works of Carnall and coworkers [97,98]. The first step comprises DFT/TDDFT, MP2/RI-CC2 or CASSCF calculations to draw the energy level diagram (Jablonski diagram) of the luminescent lanthanide complexes, including geometry relaxation, energetics and nature of the ground (singlet) and excited states (singlet and triplet) as well as to estimate the rates of all relevant radiative (fluorescence, phosphorescence) and nonradiative relaxation processes (intersystem crossing (ISC)), which involve low-energy singlet and triplet excited states. In the next step, the resonance energy transfer rates from S_1_ or T_1_ states to the acceptor excited states of Ln^3+^, the Judd–Ofelt intensity parameters, as well as the quantum efficiency and quantum yield of the lanthanide emission are evaluated [92,99,100].

The DFT (functional and basis set) and semiempirical methods are validated with respect to geometrical parameters, vertical excitation energies, emission energies and luminescence quantum yields by comparison with the experimental structural (X-ray diffraction structure) and spectroscopic parameters (UV–vis, fluorescence and phosphorescence) in solution and the solid state or with high-level ab initio methods such as MP2(full), CC2, ab initio resolution-of-the-identity algebraic diagrammatic construction through second-order (RI-ADC(2)) and the multireference configuration interaction method (DFT-MRCI) of Grimme and Waletzke [101]. The theoretical methodology should also rely on the computational methods that provide reliable energies and characterization of Ln-ligand bonding, electron density distributions in the ground (S_0_) and excited (S_1_, T_1_) states and long-range charge transfer excitations [102] and that overcome problems such as the “delocalization error” [103] and triplet instability [104]. The main challenge in the quantum mechanical treatment of lanthanides is the relativistic effects, degeneracy and the spin–orbit effect of the f orbitals, and the DFT formalism allows inclusion of part of the relativistic effects through commonly used approaches: zero-order regular approximation (ZORA) [105], Pauli approaches [106] and relativistic effective core potentials (RECPs) [107]. For the structural description of the complexes with 4f elements, it is acceptable to leave the 4f electrons in the core [108,109]. In line with the nonparticipation of the 4f electrons in the bonding, no effect of the spin–orbit (SO) coupling on the equilibrium geometries of Ln^3+^ complexes was found [110]. The TDDFT/4*f*-in-core ECP approach is a reasonable tool to examine the excitation, fluorescence and phosphorescence processes of lanthanide complexes, which mainly involve the antenna chromophore, whose electron density is insignificantly perturbed by the 4*f* electrons. One of the most widespread and proven theoretical approaches for Ln^3+^/Ln^2+^ is the relativistic effective core potential (RECP) (small and large) optimized by the Stuttgart–Dresden group with the corresponding valence basis set [111]. For example, the ECP treats [Kr]4d^10^4f^5^ (Sm(III)), [Kr]4d^10^4f^6^ (Eu(III)) and [Kr]4d^10^4f^8^ (Tb(III)) in the optimized core potential and explicitly considers the valence 5s^2^5p^6^6s^2^5d^1^6p^0^ shells. Therefore, ligand-Ln CT and Ln-ligand CT can be described by the large ECP method for Ln(III), since the valence lanthanide-centered orbitals—low-lying unoccupied (5d) and high-lying occupied (5d/6s/6p), respectively—participate in the transitions. The localized *f* electrons are energetically lower than the valence 5d/6s electrons and the probability of their participation in the transition is negligible.

On the basis of methodological studies, the long-range-corrected hybrid DFT functional, ωB97XD, with dispersion correction appears to be suitable for describing the predominant electrostatic Ln^3+^-ligand bonding in the ground state, demonstrating excellent structural performance, especially for the Eu/Sm/Gd/Tb-N/O/S bond lengths, compared to those of previously applied DFT functionals [48,80,81,82,83,110,112,113] as well as correctly locating of the triplet states. The calculated bond lengths are slightly longer, up to 0.4%. Some of the discrepancies found in the structural data may be due to the different environment in the model complexes (vacuum) and in the crystal structure (packing effects). The semiempirical RM1 and PM7 methods most accurately provide the Eu/Gd–N(phen) bond lengths (dev. 0.01–0.02 Å). It should be noted that the estimated accuracy of the DFT methods is transferable, whereas the accuracy of the semiempirical methods varies for the different lanthanide complexes depending on the specific parameterization. The low orbital overlaps and triplet instability problems in TDDFT calculations are overcome by employing the Tamm–Dancoff approximation for the triplet states [114].

The luminescent properties of lanthanide complexes are sensitive to the coordination polyhedron and shape around Ln^3+^, and for their precise description, it is useful to apply continuous shape measurements [48,115]. The luminescent Sm(pdtc)_3_(phen) complex corresponds to the specific formula M(AA)_3_(BB) and belongs to the geometric shape of a biaugmented trigonal prism (BTPR-8). It has 32 stereoisomers: 30 (15 pairs of enantiomers) in the C_1_ point group and 2 (1 pair of enantiomers) in the C_s_ point group, predicted using the Complex Build program [116]. According to the energy calculations (RM1 method), the possible stereoisomers for the lanthanide complex can be reduced to 12 (not counting the enantiomers) within which the relative enthalpy of formation (ΔH_f_) is up to 1.7 kJ/mol, and the structural parameters tolerate very small variations. The modifications (structural and energetic) of low-energy stereoisomers were estimated to have very little influence on the spectroscopic and photophysical properties studied in solution.

The influence of the surroundings on the sensitization mechanism in luminescent lanthanide complexes in solution [81,82,83], the solid phase [48] or incorporated into a silicate matrix [84] has been evaluated by simulating the environment for structure and properties close to the experimental environment. The generally accepted approaches to reproduce the solvent effect on the geometry of the lanthanide complex in the ground (S_0_) and excited states (S_1_, T_1_) are (i) micro-solvation (solvent cluster models that account for the specific solute–solvent interaction), (ii) global solvation using the polarizable continuum model and its variations (integral equation formalism variant (IEFPCM), IPCM and SMD) [117] and the conductor-like screening model (COSMO), where the solute molecule forms a cavity within the dielectric continuum of permittivity ε that represents the solvent [118] and (iii) micro-solvation and global solvation [89]. The crystal structures of the lanthanide complexes are successfully modeled using the periodic projector-augmented wave (PAW)/DFT approach. Electron exchange−correlation interactions are treated using the generalized gradient approximation as parametrized by Perdew, Burke and Ernzerhof (PBE functional) [119]. A number of luminescent Eu^3+^-based complexes have been incorporated into silica matrices for better mechanical stability, thermal stability and photostability and higher luminescence quantum yields than those of the respective pure complexes [120]. For the reconstruction of SiO_2_/Eu(phen)_2_-based materials, a suitable siloxane model was used based on a hydrophilic (Si-O-Si-OH) and a hydrophobic (Si-O-Si(CH_3_)_3_) silicate surface and adsorbed Eu(Phen)_2_X_3_ (X = Cl^−^,NO_3_^−^) complexes (Figure 6) [84]. Because of the large studied system, the semiempirical Sparkle/AM1 approach yielded reasonable optimized geometries for all the initially defined model systems. The influence of the environment on the structural and energy parameters of lanthanide complexes can be understood through a comparative analysis with the isolated complexes in the gas phase.

*Vertical excitation energy and energy of relaxation calculations.* With the development of new exchange-correlation functionals and the inclusion of complex phenomena in the time-dependent formalism, the TD-DFT method has become suitable for reproducing the electronic excited-state (EES) geometry and vertical excitation energies (VEEs), having local and charge–transfer character [121]. For both local n → π* and π → π* states, global hybrids such as B3LYP, PBE0 or M06 were found to provide accurate estimates whereas for charge-transfer or Rydberg EES, range-separated hybrids (CAM-B3LYP or ωB97xD) were recommended. The long-range corrected hybrid density functional ωB97xD [122], which includes of Grimme’s D2 or D3 dispersion corrections [123], has proven suitable for the accurate description of the energy and character of electronic transitions in different lanthanide complexes [48,80,81,83]. The gas phase VEE calculations using this method were validated against DFT-MRCI and high-level RI-ADC(2) calculations [83]. The ωB97xD and the reference calculations predicted the same character of the S_1_ state in the gas phase, unlike those obtained with PBE1PBE ones [80,83]. The TDDFT/ωB97xD calculations overestimated the S_0_ → S_1_ electron transition by ~0.3 eV (the absorption band was blueshifted by ~25 nm) as compared to the experimental absorption bands. It was established that this deviation was due to the neglect of the vibronic contributions to the excitation energies. The vibrationally resolved S_0_ → S_1_ absorption, S_1_ → S_0_ emission (fluorescence) and T_1_ → S_0_ phosphorescence spectra of Ln^3+^ complexes could be calculated within the framework of time-independent (TI) [124] and time-dependent (TD) formalisms and required optimization and harmonic frequency calculations for ground and excited states. These calculations were implemented within the framework of Franck–Condon, Herzberg–Teller and Franck–Condon–Herzberg–Teller, Duschinsky matrix rotation and sum-over-states (SOSs) approaches. The representation of the potential energy surface of the final state was achieved with adiabatic (the initial and final states were evaluated as harmonic potential energy surfaces) or vertical (the final state was evaluated around the equilibrium geometry of the initial state) Hessian models. The temperature effects at 298.15 K on one-photon absorption spectra and at 77 K on phosphorescence spectra were also included in the vibronic calculations [48,80,81,83].

For TDDFT calculation in solution, the solvation models such as the polarizable continuum model (PCM) handle solvent–solute interactions via two formalisms—the linear-response (LR) [125] or the state-specific (SS) approach [126]. The LR, which responds to the transition density between the ground and excited states, is applicable to localized excitations, whereas SS accounts for the specific electron density of the excited state and is essential for accurate modeling of charge-transfer processes. Within the framework of state-specific solvation, equilibrium and non-equilibrium solvation were used for excited-state geometry optimization and vertical electronic excitations, respectively.

For a better understanding of the distinction in the diffuse reflectance spectra of powder lanthanide complexes compared with those in solution, the linear optical properties of the crystal structures were estimated by means of the calculated frequency-dependent absorption coefficient using the periodic projector-augmented wave (PAW)/DFT approach [48]. Electron exchange−correlation interactions were treated using the generalized gradient approximation as parametrized by Perdew, Burke and Ernzerhof (PBE functional) [119]. The optical parameters were assessed for incident photon energies in the range 1.8–6.2 eV (corresponding to the UV−vis spectra in the range 700−200 nm), and the electric field polarization vectors along [100], [010] and [001] directions were assigned as xx, yy and zz. The different absorption spectra in the three directions indicate that the lanthanide complexes are three-axis optical crystals in relation to UV excitations.

*Rate and life estimation of the excited-state processes.* The sensitization mechanism of luminescent lanthanide complexes proceeds through numerous excited-state processes, some of which are competitive. The calculated rate constants and lifetimes of the relaxation processes give essential information on the channel of energy relaxation through fluorescence, S_1_-T_1_ ISC, phosphorescence, luminescence and thermal deactivation processes. Upon UV irradiation, absorption occurs by the most absorptive and higher-energy S_0_ → S_n>1_ excitation, and the absorbed UV energy is rapidly released to the lower vibrational level of the S_1_ state (for a small energy difference (up to ~0.2–0.4 eV)) on the picosecond timescale (10^−12^ s) by means of a vibro-electronic mechanism (Kasha’s rule) state [127]. This statement was supported by a calculated one-step internal conversion S_2_–S_1_ lifetime of 1.2 × 10^−12^ s. In contrast, the S_1_ → S_0_ internal conversion was estimated to be slower ~10^−9^ s due to the large S_1_–S_0_ energy gap of ~3.0–4.0 eV (UV region). Therefore, although the absorption of Ln^3+^ complexes is promoted by a higher excited state, the energy relaxation proceeds through the S_1_ state in accordance with the experimental excitation spectra, showing that excitation to the S_n>1_ and S_1_ states leads to similarly intense luminescence.

Further, a very fast geometry relaxation proceeds to the minimum of the S_1_ potential energy surface with energy stabilization. The process lifetime is of 10^−10^–10^−14^ s, comparable to the period of a molecular vibration and faster than the energy-transfer processes [128]. The large energy stabilization and geometry reorganization of the S_1_ state lead to a large Stokes shift. At this point, several scenarios are possible: the system could undergo an ISC transition to a triplet excited state T_n_, return to the ground state (S_0_), either by a radiative or nonradiative mechanism or the energy could be transferred from the ligand S_1_(D*) to Ln (A*). Accounting for the ZPE correction to ΔE_ad_(S_0_(min)–S_1_(min)) energy (TDDFT/ωB97xD method) shows a decrease in ΔE_ad_ by ~0.1 eV. The inclusion of entropy is less important for the ΔE value [81].

The energy and character of the S_1(min)_ state (nπ* and ππ*) and the oscillator strength of the S_1_ → S_0_ transition determine the values of radiative (*k_r_*) and nonradiative (*k_nr_*) rate constants and the lifetime (*τ*) of energy relaxation (fluorescence). Previous studies have shown that the S_1_ character depends on the theoretical method used [80,129]. The environment (vacuum, nonpolar and polar solution) was found to alter the S_1_ state energy, oscillator strength, character and fluorescence lifetime. In the case of stabilization of the S_1_ state with the nπ* character in a nonpolar solvent or the solid state, the S_1_-S_0_ radiative process is suppressed and favors S_1_-T_n_ ISC or excited-state energy transfer S_1_(ligand)-Ln^3+^ [82,83]. The emission lifetimes (*τ*) of singlet states have been computed for spontaneous emission using Einstein transition probability according to Equation (2) [130]:

(2) $$\tau =\frac{1}{k_{r}}=\frac{23.05}{f_{osc}{\left(\Delta E\right)}^{2}}\;\left[ns\right]$$

where *f* is the oscillator strength and Δ*E* is the energy change, characterizing the transition (TDDFT spectral data). Radiative and nonradiative rate constants and lifetimes of S_1_ → S_0_ deactivation (fluorescence and internal conversion, respectively) were also calculated using the time-dependent approach [131], as implemented within the DUSHIN code of the MOMAP2022A (Molecular Materials Property Prediction) package [132]. For further information about the methodology and application of this formalism, one could refer to Refs. [133,134].

The lack of a ligand emission band in the fluorescence spectrum of the lanthanide complexes suggests the occurrence of the preferred singlet–triplet ISC process. The direct connection of the ligand chromophore to Ln^3+^ increases the interaction of spin and orbital angular momentum, which allows mixing of the spin and angular and orbital angular momenta of S_1_ and T_n_. The spin–orbit interaction increases in the order Sm^3+^, Eu^3+^ and Tb^3+^ according to the finding that it grows with the fourth power of the metal atomic number Z. Apart from the strength of the spin–orbit coupling between two excited states, the electronic energy gap is also an important factor for the rate of S-T nonradiative transition. A small energy gap of less than 0.3 eV implies large forward and reverse rates of ISC S_1_-T_n_ and an insufficient population of the T_1_ state. With a small displacement of the S_1_ and T_1_ minima, weak coupling is expected, where energy gap law is valid, whereas with a large displacement of the S_1_ and T_1_ minima, the coupling would be strong and the transition probabilities increase with increasing energy gap [135]. On the other hand, the different orbital nature of T_1_ and S_1_ minima also enhances the spin–orbit coupling effect due to the nπ* → ππ* transition that involves a rotation of a p-orbital at some heteroatomic center.

The quantitative expression of the nonradiative (ISC) rate constants was achieved within the framework of the Fermi golden rule, Equation (3) [136].

(3) $$k_{ISC}=\frac{2\pi }{\hbar }{\left|V\right|}^{2}\rho_{FC},$$

where |*V*| is the strength of the perturbation or the interaction between the initial and final states. For states with different parity the parameter coincides with the strength of the spin–orbit coupling (SOC) interaction between the respective singlet and triplet states. The final term in Equation (3) is the Franck–Condon-weighed density of states (FCWD), denoted by *ρ*_FC_. This parameter could be assessed on the basis of geometry optimization and harmonic frequency calculations for each of the states involved in the transition process. When the energy difference between the initial and final states is rather small, it could be assumed that *ρ*_FC_ ~ 1/ΔE. This is the case when a transition of the type S_1_ → T_n_ (n = 1, 2) is examined.

For the lanthanide complexes, the spin–orbit coupling and the intersystem crossing rates of the transitions S_1_ → T_1_, T_1_ → S_1_ and T_1_ → S_0_ were assessed on the basis of SA-CASSCF/NEVPT2 wave functions. The quasi-degenerate perturbation theory (QDPT) [137] was implemented to evaluate the spin–orbit coupling between low-lying singlet and triplet excited states as well as zero-filed splitting of the T_1_ state of the molecules. More computational details are given in refs [48,80,81].

For phosphorescent emission, an average rate constant is calculated on the basis of ${\bar{k}}_{ph}$ estimates for the particular T_nm_-states (*m* = *x*, *y*, *z*), Equation (4):

(4) $${\bar{k}}_{ph}=\frac{\sum_{m=x,y,z}k_{p}^{\left(m\right)}e^{-\frac{∆E_{m}}{k_{B}T}}}{\sum_{m=x,y,z}e^{-\frac{∆E_{m}}{k_{B}T}}};\;k_{ph}^{\left(m\right)}=\frac{{\left(∆E_{m}\right)}^{2}f^{\left(m\right)}}{23.05}\;\left[{ns}^{-1}\right];\;f^{\left(m\right)}=\frac{2}{3}{\left|\left\langle \Psi_{0}^{SOC}|\hat{\mu }|\Psi_{m}^{SOC}\right\rangle \right|}^{2}∆E_{m}.$$

Here, *k_ph_*^(^*^m^*^)^ is the rate of phosphorescence emission for an individual *T_n_*—triplet level, *k*_B_ denotes the Boltzmann constant, *f*^(^*^m^*^)^ is the oscillator strength, $\hat{\mu }$ is the dipole moment operator, while $\left.\left|\Psi_{0}^{SOC}\right.\right\rangle$ and $\left.\left|\Psi_{m}^{SOC}\right.\right\rangle$ are the wavefunctions of the mixed ground and *m*-th triplet excited state. It should be noted that the right-hand side of Equation (4) is correct if the emission energy Δ*E* is evaluated in atomic units, while the transition dipole moment value is taken in length representation.

It should be mentioned that similar T_1_ energy levels were calculated for the ligand chromophore and the corresponding homoleptic Ln^3+^ complexes. In this case, the T_1_ state energy of Ln^3+^ complexes could be derived from the experimental phosphorescence spectra of the ligand and the corresponding Gd^3+^ complexes. The population of the triplet state and the theoretical phosphorescence quantum yield of the ligand and its Ln^3+^ complexes should, however, be different because of the contribution of the heavy metal to the strength of the spin–orbit coupling between T_1_ and S_0_.

*Theoretical approach to luminescence: LUMPAC.* The radiative and nonradiative energy transfer from S_1_ or T_1_ donor states of the ligand to the acceptor levels of Ln^3+^ would contribute to the luminescence emission. The radiative energy transfer process is considered to be less efficient because only a small fraction of the emitted light can be captured by the acceptor. The more probable nonradiative energy transfer is described by two mechanisms: electrostatic multipolar (Förster) and double-electron exchange (Dexter). The energy transfer from the singlet state is operated by (i) the Förster resonance mechanism, involving dipole–dipole interaction and long-range interactions (up to ~30–100 Å, with a distance dependence of 1/r^6^) or (ii) the Dexter mechanism, known as exchange (short-range donor–acceptor distances, with a distance dependence of e^−r^) [138]. The triplet energy transfer is explained only according to the Dexter mechanism [139]. Therefore, the total rate for energy transfer can be better described as k_ET_ (total) = k_ET_ (Förster) + k_ET_ (Dexter) by the theoretical model developed in the literature [57].

The resonance ligand-to-metal energy transfer rates were calculated following the approach developed by Malta and coworkers [140]. First, the intensity parameters Ω_λ_ (λ = 2, 4) and the radiative deactivation rate of ^5^*D*_0_ (the key emissive state of Eu^3+^) were extracted from the experimental emission spectra of the Eu^3+^ complexes and lifetime of the lanthanide emission. Subsequently, theoretical estimates of the same values were obtained following the method proposed by de Sá et al. [96] and extended by Freire and colleagues (QDC scheme) [99]. Finally, the intensity parameter values and the rates of deactivation were used in the procedure for the theoretical estimation of the energy transfer rates L(S_1_/T_1_) → Ln and the luminescence quantum yield and efficiency.

The question arises of how to combine DFT/TDDFT and semiempirical methods, as well as the Judd–Ofelt and Malta approaches, to describe the complete photophysical process from absorption to luminescence for the lanthanide complexes. The latest approach for calculating the rate of ligand-to-lanthanide energy transfer uses ligand-centered vertical excitations. The optimized geometry can be (i) in the ground state, obtained using the semiempirical RM1 method or the DFT method, or (ii) in the excited states (S_1_/T_1_), obtained using the TDDFT method. Further, vertical excitation energies could be calculated by the INDO-S/CIS or TDDFT/TDA methods. The main requirement for a reliable estimation of the energy transfer rate to the potential donor states of Eu^3+^ (^5^D_0_, ^5^D_1_) is that the calculated energy level of the donor state T_1_ (according to the methods mentioned above) matches the energy level of the TDDFT triplet state minimum and, therefore, the experimental emission from T_1_. The motivation for using the T_1_ energy level, obtained at the TDDFT level (T_1_ geometry optimization and VEE) comes from the fact that in many cases, there are distinctive differences between the structural parameters in the ground and excited states. At the same time, the energy transfer process is considered to be fast enough that no changes in molecular geometry are expected. The calculations also showed a similar geometry of the S_0_ and T_1_ states. Therefore, a more accurate evaluation of the energy transfer rates from a certain ligand-centered excited singlet or triplet state would involve intensity parameters and wavefunctions calculated at the energy minimum of that state. While LUMPAC allows the use of both semiempirical and TDDFT wavefunctions, it is our observation that the rate estimates based on first principles tend to be too high, leading to values of 10^11^ s^−1^ and higher. As a consequence, INDO-S/CIS wavefunctions [141] are considered more compatible with the computational scheme.

*Theoretical prediction of excited-state energy mechanism in luminescent lanthanide complexes with N- and O-donor ligands.* The advanced computational strategy combining DFT/TDDFT and multireference ab initio calculations with perturbative spin–orbit coupling corrections and the Judd–Ofelt and Malta theories and the QDC model in conjunction with experimental data allowed a deeper understanding of the role of the antenna chromophore and the sensitization mechanism in luminescent lanthanide complexes: 1,10-phenanthroline (phen) (Eu(phen)_2_(NO_3_)_3_ and Tb(phen)_2_(NO_3_)) [80,82,84], coumarin-3-carboxylic acid (HCCA) (Eu(CCA)Cl_2_(H_2_O)_2_, Eu(CCA)_2_Cl(H_2_O)_2_, Eu(CCA)_3_(H_2_O)_3_, Tb(CCA)_2_Cl(H_2_O), Tb(CCA)_2_(NO_3_)(H_2_O)) [83], phenacyldiphenylphosphine oxide (Phenac), Eu(Phenac)_2_(NO_3_)_3_(H_2_O), Eu(Phenac)_2_(NO_3_)_3_, Eu(Phenac)_3_(NO_3_)_3_ and Tb(Phenac)_2_(NO_3_)_3_(H_2_O) [81]. The photophysical properties of the ligands, their binding ability to encapsulate and stabilize lanthanide complexes, and L-M bonding strength and character have been evaluated in relation to the luminescence mechanism. The main steps of the sensitization mechanism, including absorption, relaxations, intersystem crossing, energy transfer and quantum yield, are traced in the calculated energy level diagrams of the lanthanide complexes in the gas phase and solution (polar and nonpolar).

In the homoleptic Ln(phen)_2_(NO_3_)_3_ (Ln^3+^ = Eu, Tb, Gd), *phen* is an antenna chromophore and is N,N-bidentately bonded to Ln^3+^ (Figure 7). The counterion does not participate directly in the absorption process, but can affect the energy and character of the S_1_ and T_1_ states, involved in the excited-state energy transfer. The small oscillator strength of the S_1_ state in the gas phase (or nonpolar solvent) and DMF produces a delay of the S_1_-S_0_ emission. For Tb(phen)_2_(NO_3_)_3_, the calculated shorter radiative S_1_ → S_0_ lifetime (~10^−9^ s) than that of nonradiative S_1_ → T_2_/S_1_ → T_1_ ISC lifetime (~10^−12^ s) indicates that the ISC process to the triplet state is more probable than the fluorescence [80]. The similar calculated energy gap between S_0_ and T_1_ state minima (S_0_(opt)-T_1_(opt)) for *phen* and Ln(phen)_2_(NO_3_)_3_ (Ln = Eu, Tb, Gd) complexes in the gas phase and DMF, shows that the solvent effect on the lowest triplet state is negligible, and the T_1_ state energy of the *phen* chromophore determines that in its Ln^3+^ complexes [80,82]. Two relaxation channels of ISC, S_1_ → T_2_ → T_1_ and S_1_ → T_1_, are predicted for the population of the T_1_ state based on the calculated nonradiative rates of the forward and back-transfer S_1_ ↔ T_2_, T_2_ ↔ T_1_, S_1_ ↔ T_1_ and T_1_ ↔ S_0_ processes. Further, the energy transfer proceeds from a ligand-centered donor level T_1_ to the main emissive metal-centered states, ^5^D_0_ and ^5^D_4_ of Eu(III) and Tb(III), respectively. The most effective electronic relaxation and energy transfer mechanisms were predicted: S_0_ → S_1_ → T_1_ → ^5^D_1_/^5^D_0_ for Eu^3+^ and S_0_ → S_1_ → T_2_ → T_1_ → ^5^D_4_ for the Tb^3+^ complex. The other possible excitation channels S_1_ → ^5^D_2_/^5^L_6_ (for Eu^3+^) and S_1_ → ^5^D_3_ and T_2_ → ^5^D_3_ (for Tb^3+^) were found to be less probable. It was established that *phen* acts as a better antenna for Eu^3+^ than for Tb^3+^. A possible explanation is that the transfer from the T_1_ state could populate two possible Eu^3+^-centered acceptor states: ^5^D_0_ and ^5^D_1_. The energy differences ΔE(T_1_ → ^5^D_0_) ~ 0.64 eV, and ΔE(T_1_ → ^5^D_1_) ~ 0.42 eV are quite large compared to ΔE(T_1_ → ^5^D_4_) ~0.24 eV for the Tb^3+^ complex. At such large ΔE, reverse transfer rates ^5^D_0_ → T_1_ and ^5^D_1_ → T_1_ are low and the overall energy conversion efficiency is higher for the Eu^3+^ complex (QY of 35%, calc. 32%) than that of the Tb^3+^ complex (13%). The process in the Eu^3+^ complex is governed by the exchange mechanism, and the main observed emission is known to be at a maximum at 615 nm due to the ^5^D_0_ → ^7^F_2_ transition and is responsible for the red emission color. The spectrum of the Tb^3+^ complex is dominated by the ^5^D_4_ → ^7^F_5_ transition, located at 541 nm, which is responsible for the green emission color.

The structural and photophysical properties (Jablonski diagrams and nonradiative rates) of two highly emissive Eu^3+^-based luminescent complexes, Eu(Phen)_2_Cl_3_ and Eu(Phen)_2_(NO_3_)_3_ adsorbed on hydrophilic Si-O-Si-OH and hydrophobic Si-O-Si(CH_3_)_3_ silicate surfaces were elucidated by combined semiempirical(Sparkle/AM1; INDO/S-CIS) and simplified TDDFT calculations (Figure 6) [84]. The calculated data explained the highest luminescence quantum yield (experimental and theoretical) of Eu(Phen)_2_(NO_3_)_3_ incorporated in hydrophilic matrix compared with that in a hydrophobic one. For Eu(Phen)_2_Cl_3_, the observation of a large number of hydrogen bonds on the hydrophobic silicate surface and the strong adsorption interaction appear to be operative factors that suppress the nonradiative emission and contribute to the higher luminescence quantum yield of the complex incorporated into a hydrophobic matrix than that incorporated into a hydrophilic one. The calculated energy differences ΔE = ((E(T_1_) − E(^5^D_0_)/E(^5^D_1_)) varied in the range ~0.5–0.7 eV and they were large enough to favor the T_1_-^5^D_0_ and T_1_-^5^D_1_ energy transfer relative to the back energy transfer (Table 5). As the energy band gap ΔE(T_1_-^5^D_0_) increased from 0.47 to 0.66 eV, the quantum efficiency and quantum yield became larger, but if it exceeded 0.7 eV, η and QY decreased dramatically. The theoretical research revealed other operative factors for the formation of the luminescence quantum efficiency and yield of Eu(Phen)_2_X_3_ when incorporated into SiO_2_-based matrices.

The competitive excited-state processes in the homoleptic Eu^3+^ and Tb^3+^ complexes of coumarin-3-carboxylic acid (HCCA)—fluorescence, intersystem crossing (ISC) and phosphorescence, were analyzed with respect to the environment, number of the ligands (1–3), Ln^3+^ ion type (Eu and Tb) and counteranion (Cl^−^ and NO_3_^−^) (Figure 8) [83]. The HCCA is bidentately bonded to Ln(III) through two oxygen atoms. It was found that the environment altered the S_1_ state energy, oscillator strength, fluorescence lifetime as well as the S_1_ character—polar solution stabilized the S_1_(ππ*) state, whereas the nonpolar solution (gas phase, solid state) stabilized the S_1_(nπ*) state. The S_1_(nπ*) state, characterizing by small oscillator strength, was a decisive factor for the resulting excited-state energy transfer and Ln(III) luminescence. It suppressed the S_1_ ligand emission in the complexes and favored S_1_-T_1_ ISC or direct transfer to the emitting levels of Ln^3+^. The HCCA triplet (T_1_) state energy and ππ* character were preserved in the Eu/Tb-CCA complexes and are not changed by the environment. The energy gap between the higher-energy T_1_ donor state and the acceptor levels ^5^D_1_ of Eu^3+^ (~0.5 eV) and ^5^D_4_ of Tb^3+^ (~0.1 eV) provided optimal resonance conditions for effective energy transfer to Eu^3+^, but a lower probability for Tb^3+^. The excitation channel T_1_ → ^5^D_0_ through an exchange mechanism was predicted as the most probable one to populate the main emissive Eu-centered state in the complexes. The energy and the ππ* character of the populated T_1_ state were not influenced by the H_2_O ligands, the Ln(III) type, the counterion type and the environment. The higher luminescence quantum yield of Eu(CCA)_3_(H_2_O)_3_ compared to Eu(CCA)_2_Cl(H_2_O)_2_ (from experimental and theoretical points of view) was explained by the higher Eu^3+^ radiative emission rate, the lower nonradiative decay rate, the shorter distances (LR) between the singlet and triplet CCA donor states and the Eu^3+^ acceptor states and the higher refractive index (solid state).

The sensitization capacity of the *Phenac* ligand, combining C = O and P = O donor groups, was theoretically explored in the Eu^3+^ and Tb^3+^ complexes in vacuum, solution and the solid phase. The influence of the coordination polyhedron (number of *Phenac* ligands), ligand bonding type (mono- and bidentate) and strength, the environment, the characteristics of the emissive singlet and triplet states that stimulate the Eu^3+^/Tb^3+^ luminescence was elucidated to address the open question in Ref. [142] about the type of Eu^3+^ complexes formed in solution. The thermodynamic stability calculations for Eu(Phenac)_2_(H_2_O)(NO_3_)_3_, Eu(Phenac)_2_(NO_3_)_3_ and Eu(Phenac)_3_(NO_3_)_3_ in reactions predicted that Eu(Phenac)_3_(NO_3_)_3_ was mainly stabilized in solution and the gas phase, while the other two complexes were present at lower concentrations [81]. Similar relaxation paths were found for all complexes (Figure 9). The energy absorbed by S_n>1_(π-π*) states relaxed to T_1min_ via the major ISC mechanism S_1_ → T_2_ → T_1_. The long radiative lifetime of S_1_ → S_0_ due to n(p(C)(O))π-π*,p(P) character facilitated the forward ISC process. The environment, number of Phenac ligands and Ln^3+^ size affected the T_1_ energy by ~0.1 eV. The calculated (Phenac → Eu^3+^ energy transfer rates suggested the most likely T_1_ → ^5^D_1_/^5^D_0_ channels for the population of emitting metal-centered states. The calculated quantum efficiency of energy transfer through the T_1_ state was consistent with the experimental value (Table 5). The lower experimental quantum yield (~4%) in solution compared to the calculated value (~39%), however, was due to insufficient electron population of the T_1_ donor state resulting from the high reverse rate of the T_1_ → S_0_ process and the large energy difference ΔE(T_1_-^5^D_1_/^5^D_0_), which exceeds 0.7 eV for Eu(III) compared to the optimal energy transfer range of 0.3–0.6 eV. The higher luminescence quantum yield (experimental and theoretical) for the Tb^3+^-Phenac complexes was explained by the lower T_1_ state of 0.1 eV and the more suitable ΔE(T_1min_ − ^5^D_4_) of ~0.5 eV for energy transfer as well as the possible enhancement of spin–orbit coupling effects by the heavier Tb^3+^ compared to Eu^3+^.

## 6. Conclusions

The present review summarizes the most efficient theoretical approaches for studying a broad range of transition metal clusters and complexes of the 3d elements as well as the f-block elements. The role of sulfide and chalcogenide ligands is elucidated: in the 3d elements they favor photochemical activation of the core, and other electron-donor ligands such as carbonyl groups contribute to thermodynamic stability and favor a shift of light absorption bands to the visible region. DFT-calculated energy profiles of the chemical reactions of water splitting and carbon dioxide reduction provide accurate activation barriers and reaction enthalpies. As the complexes of M_2_X_2_ (M = Fe, Co, Ni, Cu; X = S, Se) mimic both hydrogenase and carbon monoxide dehydrogenase enzymes (CODH) the reactions of hydrogen evolution (HER), oxygen evolution (OER) and the two-step carbon dioxide reduction to formic acid/CO + H_2_O have proved feasible. Lanthanides also benefit from S,S-donor ligands in sensitizing the metal center, yielding lanthanide (Eu) sulfide nanoparticles with photocatalytic properties or luminescent materials suitable for novel electronic and optical devices. A more complex energy transfer mechanism operating in the antenna effect, encompassing energetics and rates of all possible competing excited-state processes and luminescence sensitization via ligand singlet states and charge transfer states, was revealed by applying well-validated and parametrized theoretical approaches in close relation to the experiment. The discrete processes that govern photoluminescence, unusual photophysical properties and factors responsible for excited-state energy transfer in lanthanide complexes have been successfully predicted by combined DFT/TDDFT, multireference and semiempirical calculations, taking into account spin–orbit coupling and relativistic effects. The theoretical methods and approaches that reliably simulate, explain and predict transition metal systems and their catalytic and optical properties are a powerful tool for the design of new functional metal systems. The in-depth and closely related theoretical and experimental study of 3d and 4f complexes provides a powerful impetus for the further development of theoretical methods, as well as for directed syntheses and improved spectroscopic techniques.

## Figures and Tables

**Figure 1:**
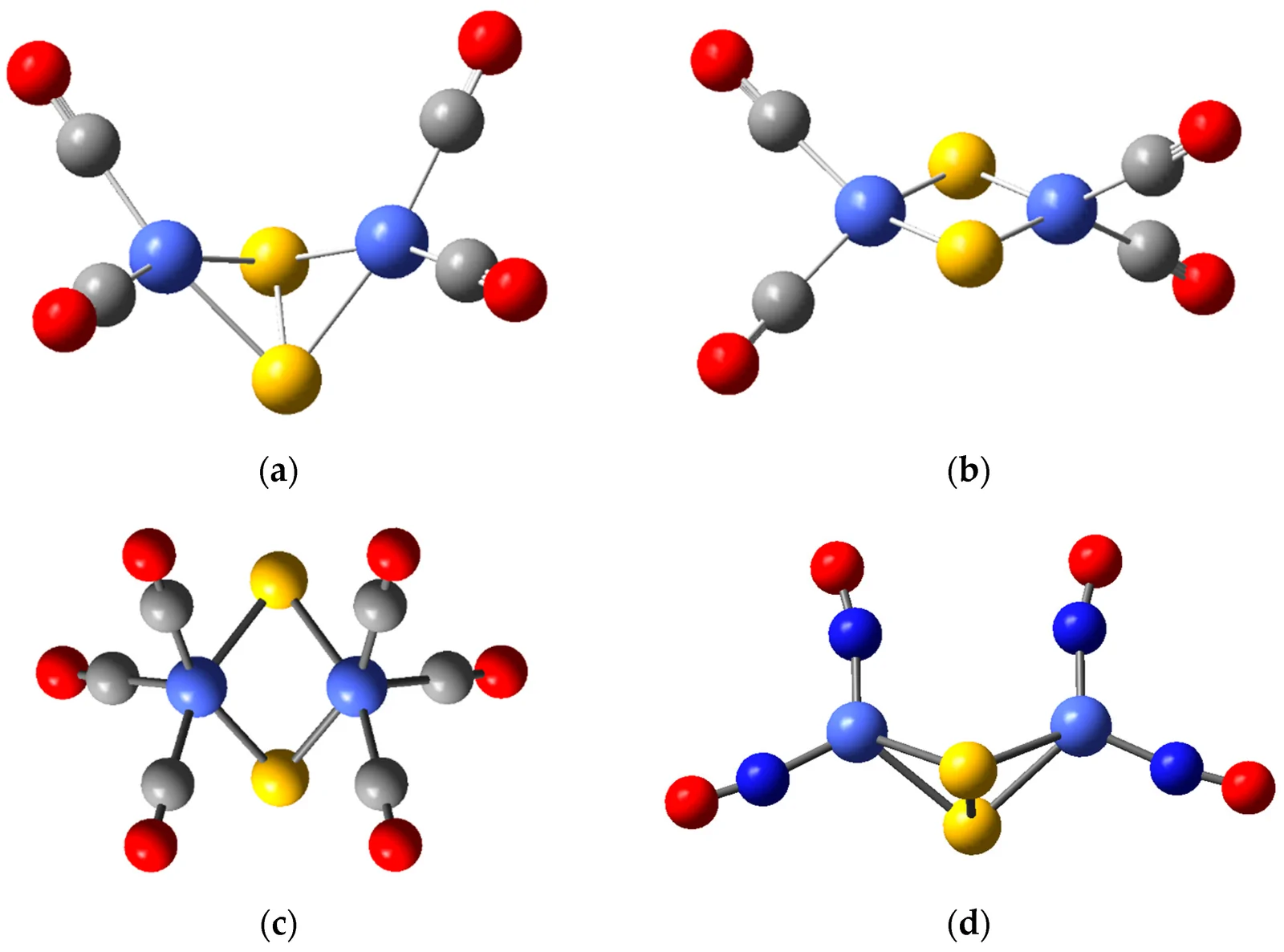
(**a**) The global minimum of Co_2_S_2_(CO)_4_ in rhombic non-planar configuration. (**b**) Co_2_S_2_(CO)_4_ with a planar core. (**c**) Co_2_S_2_(CO)_6_ in non-planar antiferromagnetic ground state. (**d**) Co_2_S_2_(NO)_4_ in non-planar diamagnetic ground state. Legend: the Co atoms are blue, the sulfur atoms are yellow, the carbon atoms are grey, the oxygen atoms are red and the nitrogen atoms are dark-blue. From refs. [21,32].

**Figure 2:**
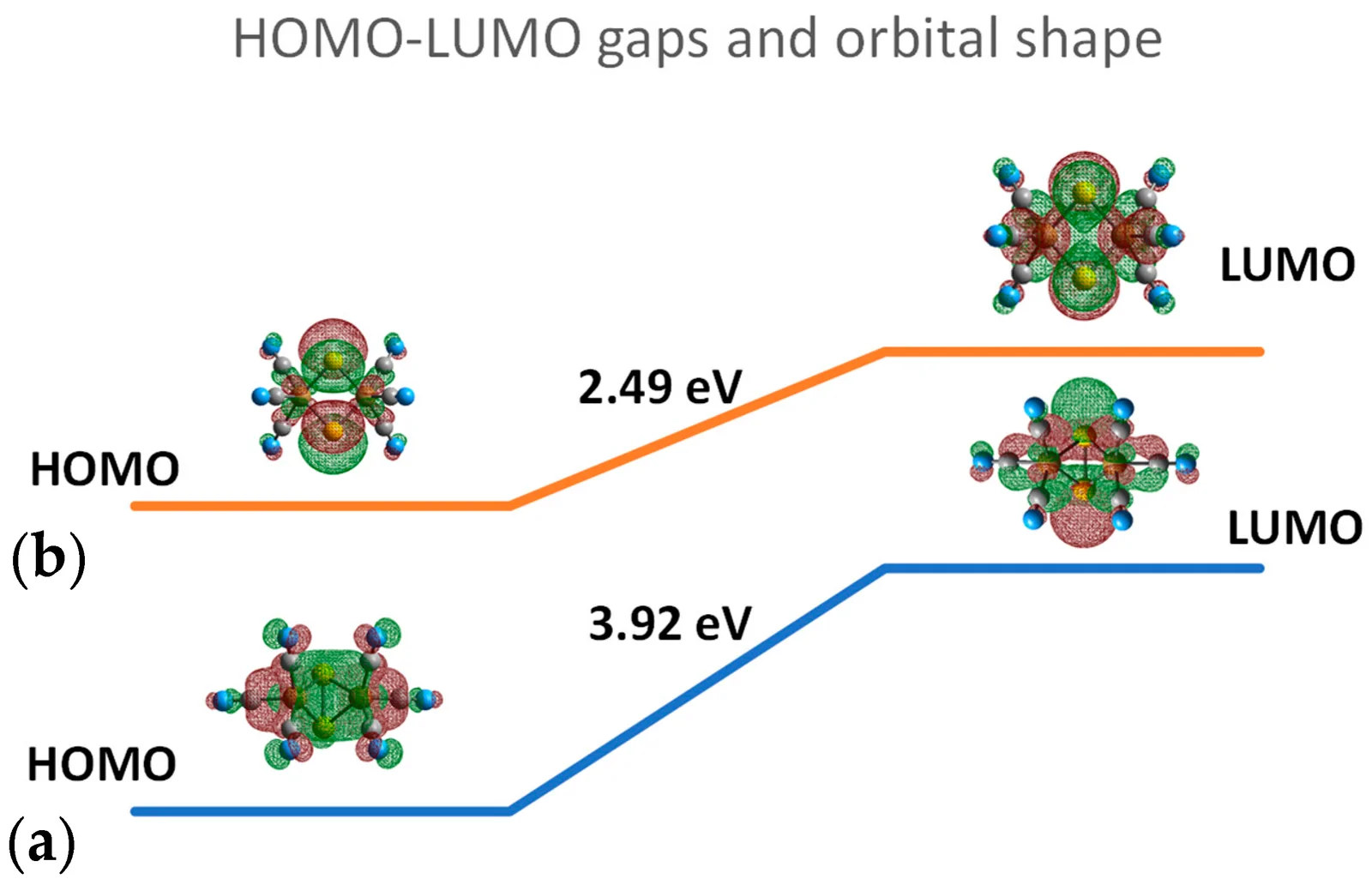
HOMO-LUMO gaps and contour maps of the HOMO and LUMO of (**a**) the Fe_2_S_2_(CO)_6_ complex with a non-planar core (the global energy minimum configuration) and (**b**) the HOMO and the LUMO of the Fe_2_S_2_(CO)_6_ complex with a planar core (low-lying conformational isomer). Legend: the positive parts of MOs are red, the negative parts are green, the iron atoms are brown, the sulfur atoms are yellow, the carbon atoms are grey and the oxygen atoms are blue.

**Figure 3:**
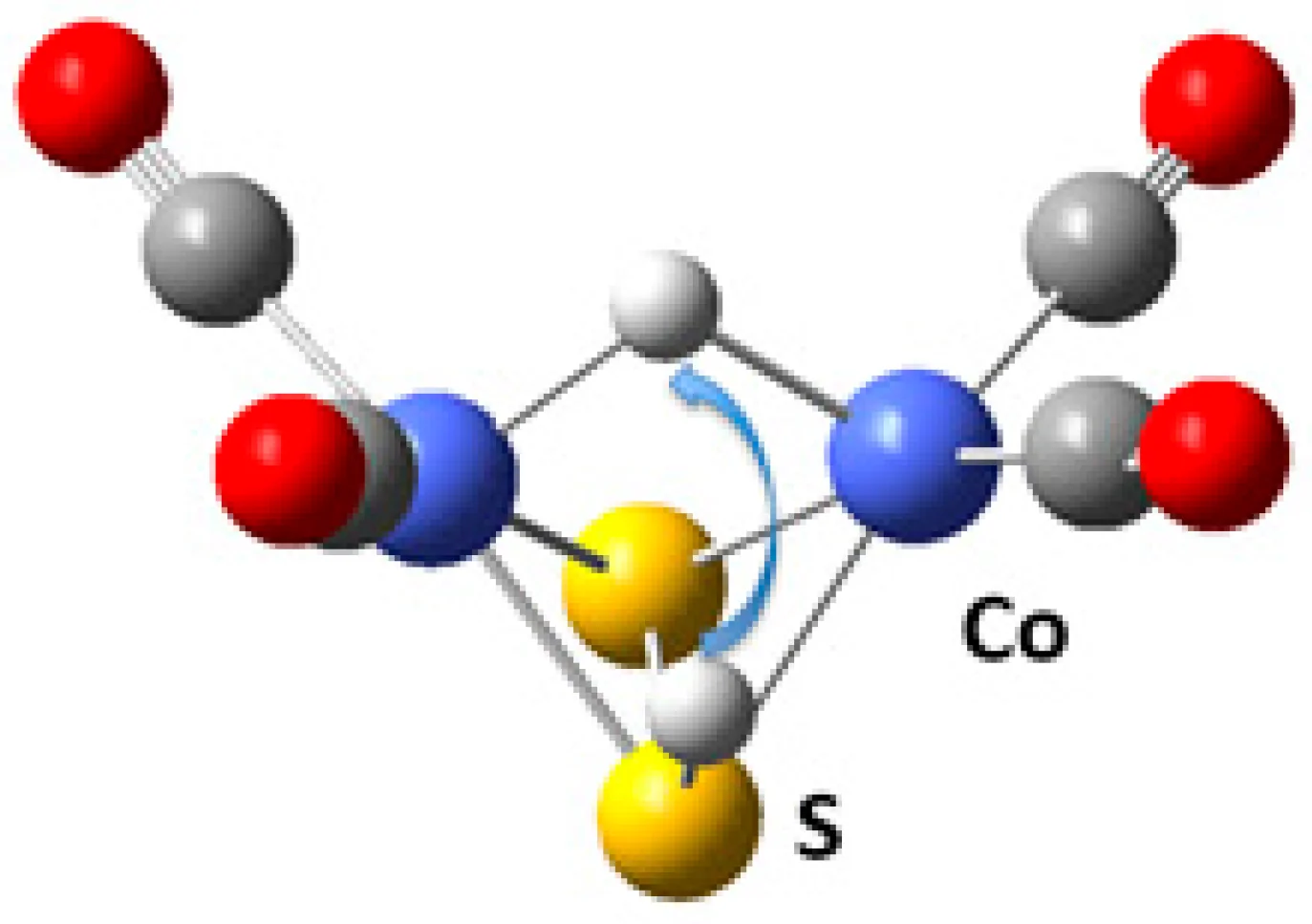
The two distinct positions of hydride in Co_2_S_2_(CO)_4_. Legend as in Figure 1; the hydrogen atoms are small grey circles, cobalt atoms are blue circles. From ref. [21]. The configuration with hydrogen coordinated midway to both Co centers lies 79 kJ mol^−1^ higher in energy.

**Figure 4:**
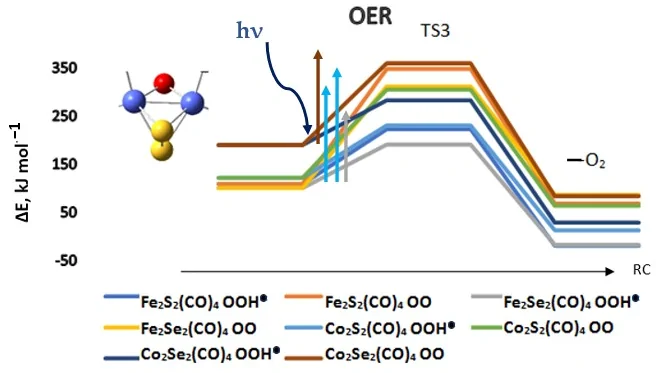
The reaction path of oxygen formation and oxygen evolution for cobalt and iron chalcogenide complexes. ΔE is the energy difference relative to the ground-state complexes; RC—reaction coordinate. The excitation energies of representative most intense transitions induced by light absorption are denoted by vertical arrows. From reference [21].

**Figure 5:**
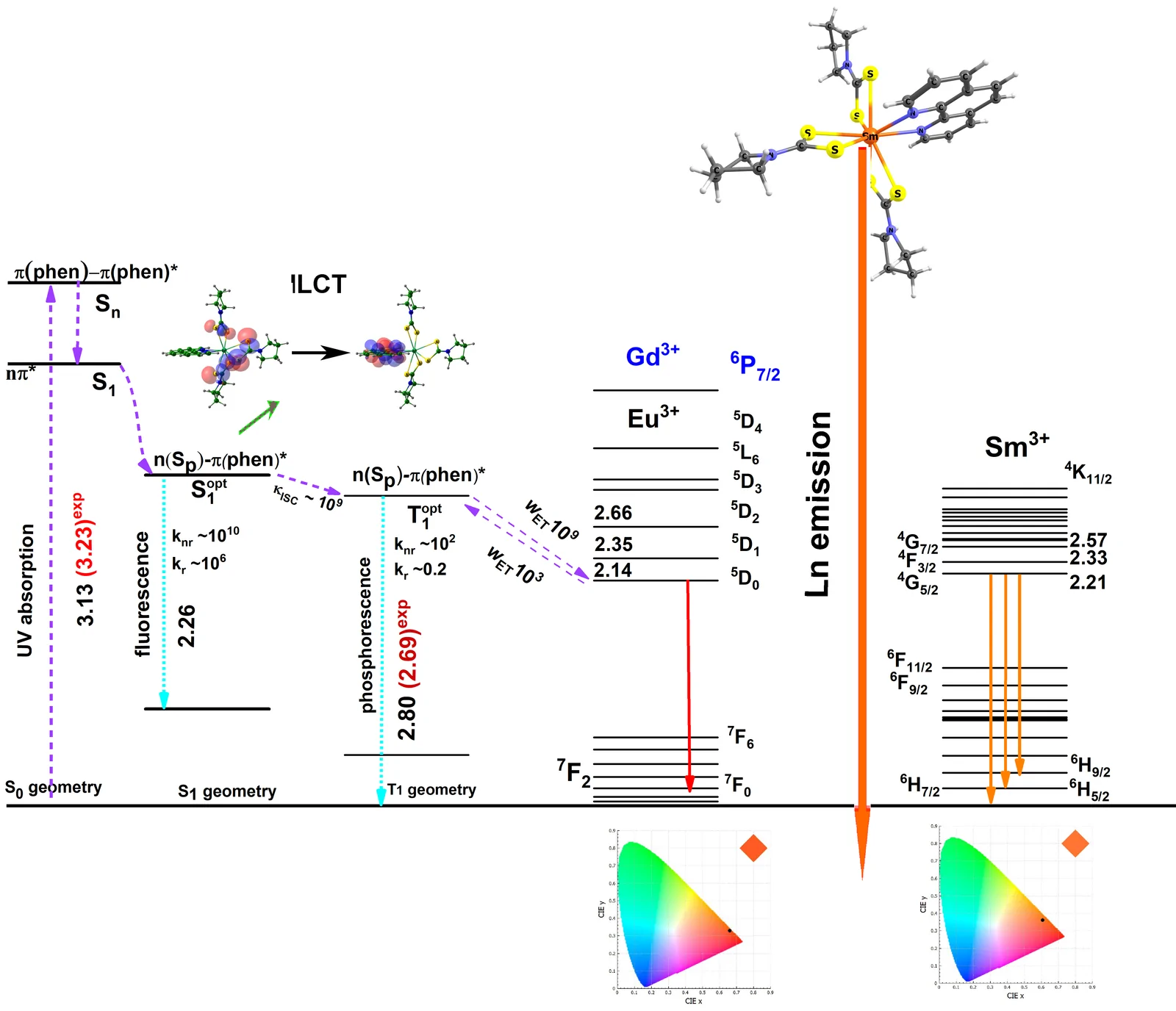
Ligand-centered energy level diagram of Ln(pdtc)_3_(phen) (Ln = Sm(III), Eu(III), Gd(III)) in DCM solution, predicted by TD-DFT/ωB97xD calculations and the most probable channel for intramolecular energy transfer in the excited state. Energy values (in eV) and rates (K_r,_ K_nr_, K_ISC_, W_ET_) (in s^−1^).

**Figure 6:**
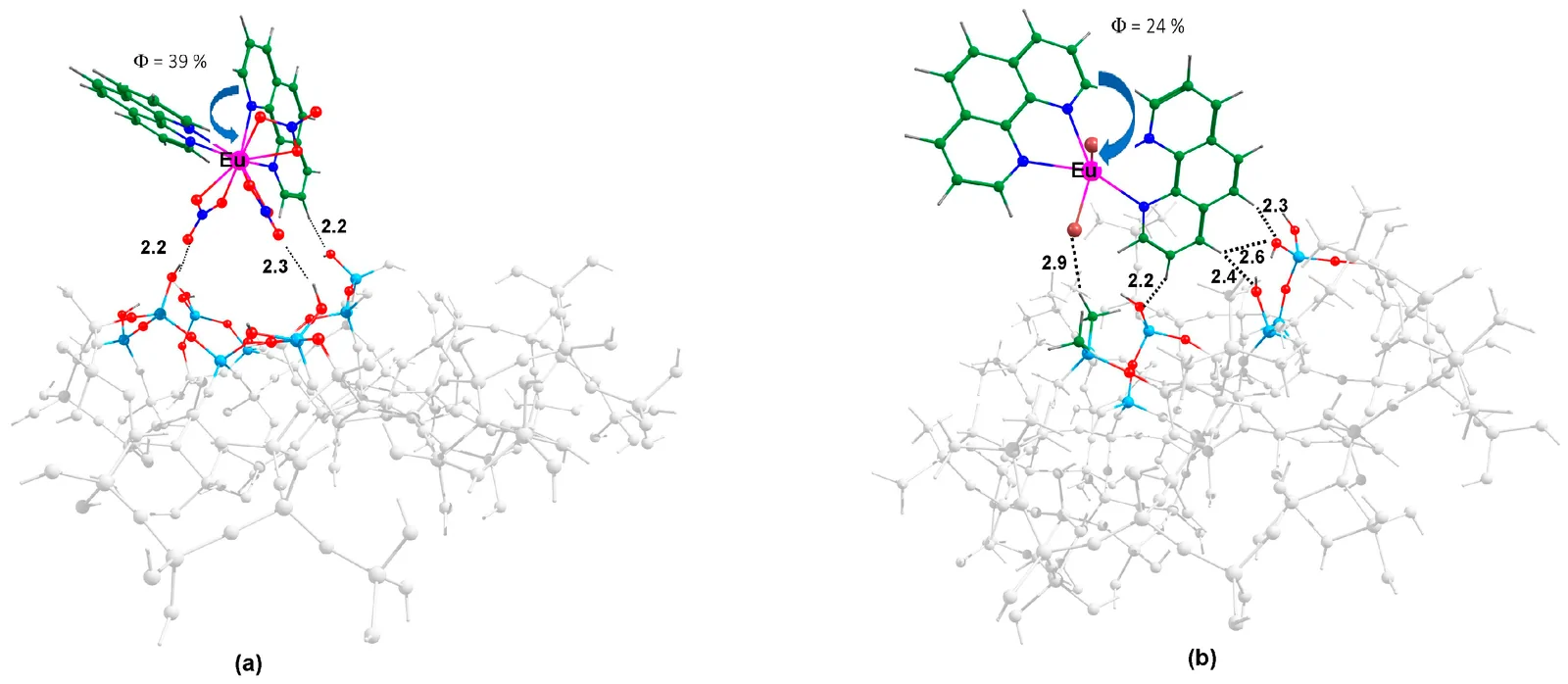
Modeling of highly luminescent Eu(phen)_2-_based complexes incorporated into silicate matrices and the decisive role of strong adsorption interaction via hydrogen bonds in suppressing nonradiative emission and enhancing the luminescence quantum yield (Φ): (**a**) Eu(Phen)_2_(NO_3_)_3_ adsorbed on a hydrophilic SiO_2_-based model-surface composite and (**b**) Eu(Phen)_2_(Cl)_3_ adsorbed on a hydrophobic SiO_2_-based model-surface composite. Atom color legend: green (carbon), blue (nitrogen), grey (hydrogen), red (oxygen), sky blue (Si), purple (Ln).

**Figure 7:**
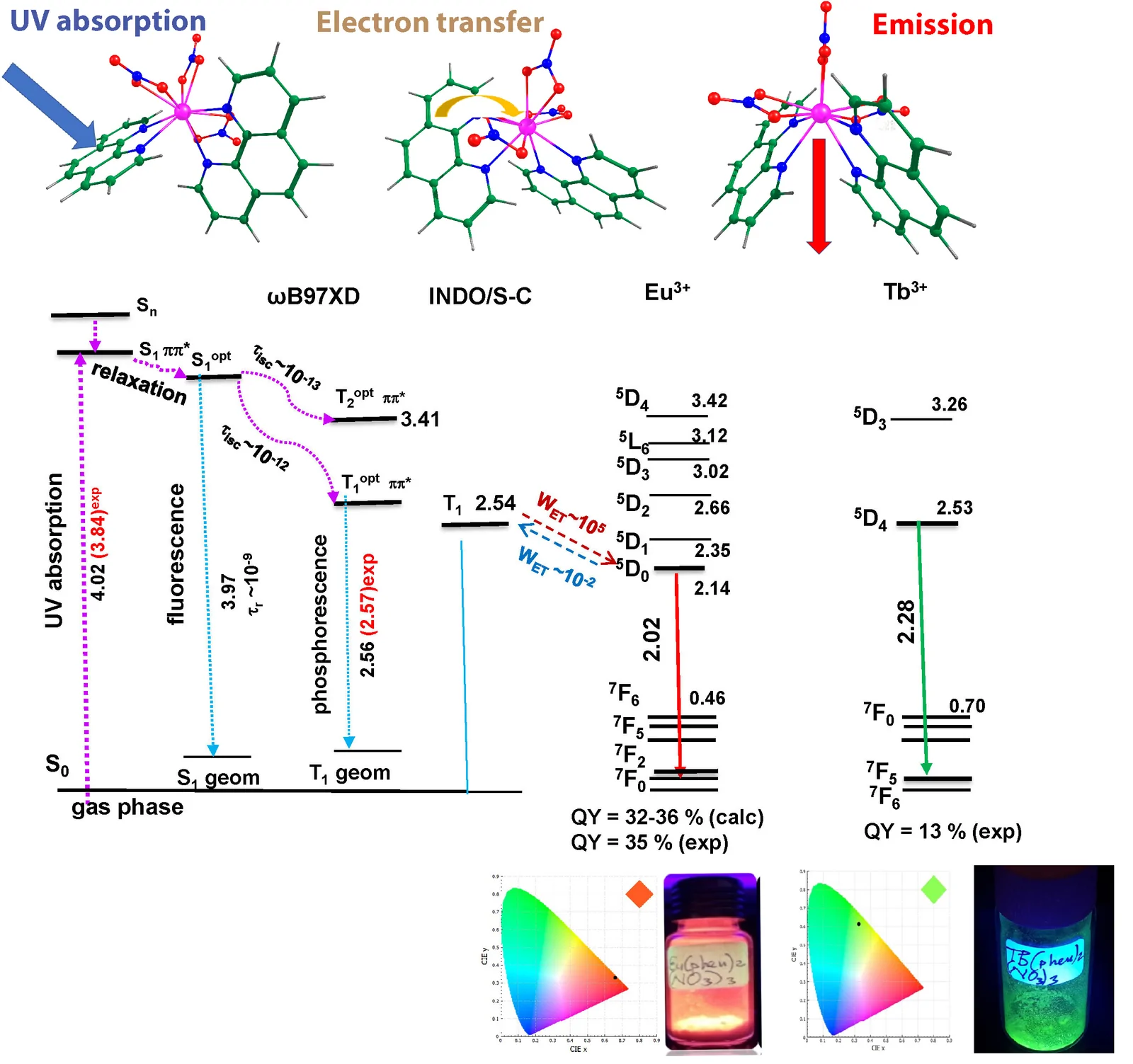
Ligand-centered energy level diagram of Ln(phen)_2_(NO_3_)_3_ (Ln = Eu(III), Tb(III)) predicted by DFT/TDDFT/ωB97xD calculations and the most probable channel for the intramolecular energy transfer process. Energy values (ΔE^vibr-el^) with vibro-electronic contribution (in eV), radiative and nonradiative lifetimes (in s), W_ET_ rates (in s^−1^). W_ET_ — L-M energy transfer rate; QE — quantum efficiency, QY — quantum yield, Atom color legend: purple (lanthanide), green (carbon), blue (nitrogen), red (oxygen), grey (hydrogen).

**Figure 8:**
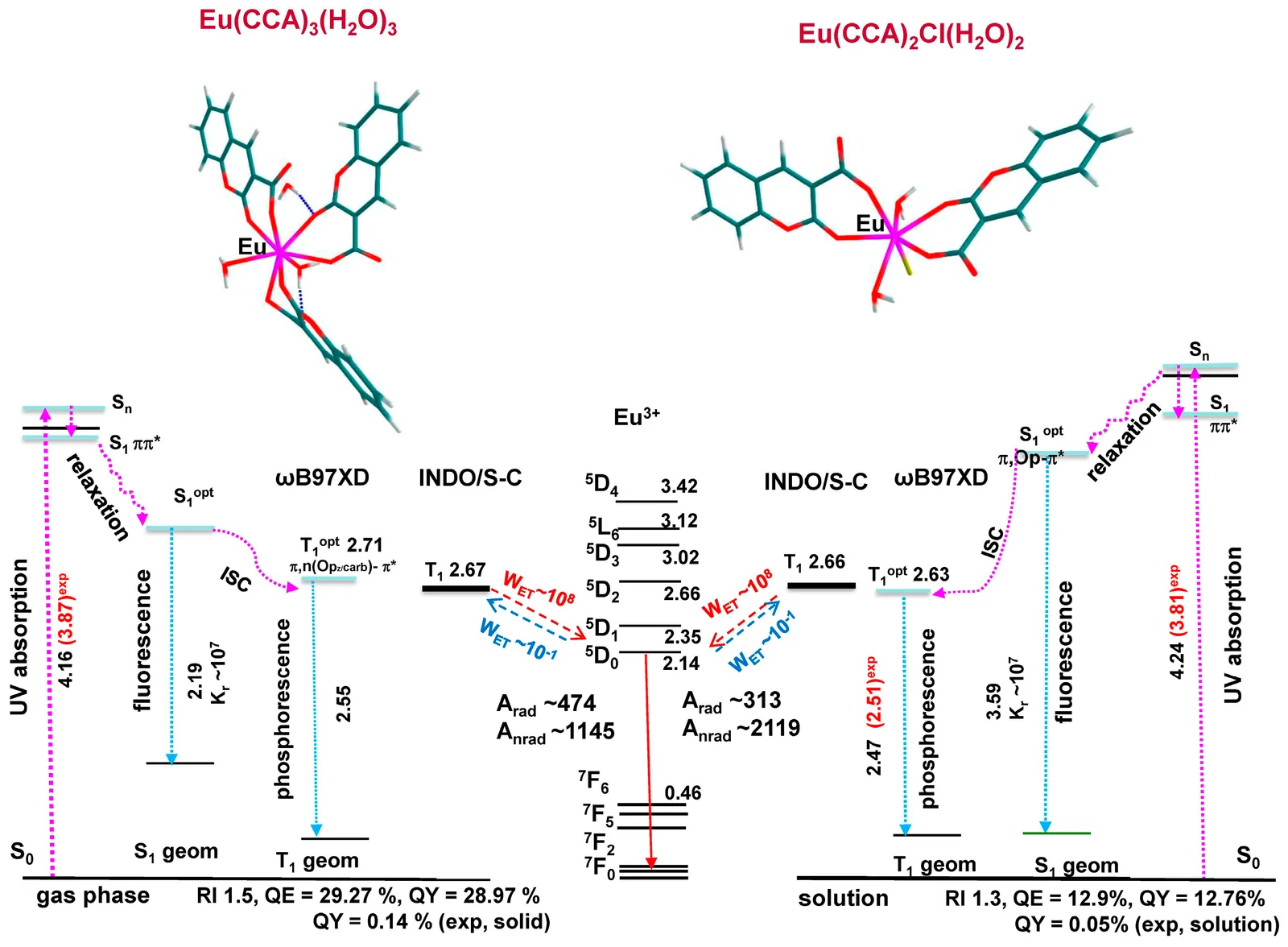
Energy level diagram calculated using the DFT/TDDFT(TDA)/ωB97XD method of Eu(CCA)_3_(H_2_O)_3_ in the gas phase (with reference to the solid state) and Eu(CCA)_2_Cl(H_2_O)_2_ in solution, compared to the available experimental data (exp) (in red). INDO/S-CIS calculations and the most probable channel for the nonradiative energy transfer process based on calculated energy transfer (red) and back-energy transfer (blue) rates (in s^−1^). ISC—intersystem crossing, k_r_—fluorescence radiative rate (in s^−1^), A_rad_ (radiative emission rate) and A_nonrad_ (nonradiative emission rate) (in s^−1^), opt—energy of optimized excited-state geometry, RI—refractive index, QE—quantum efficiency, QY—quantum yield. Atom color legend: purple (europium), red (oxygen), green (carbon), grey (hydrogen).

**Figure 9:**
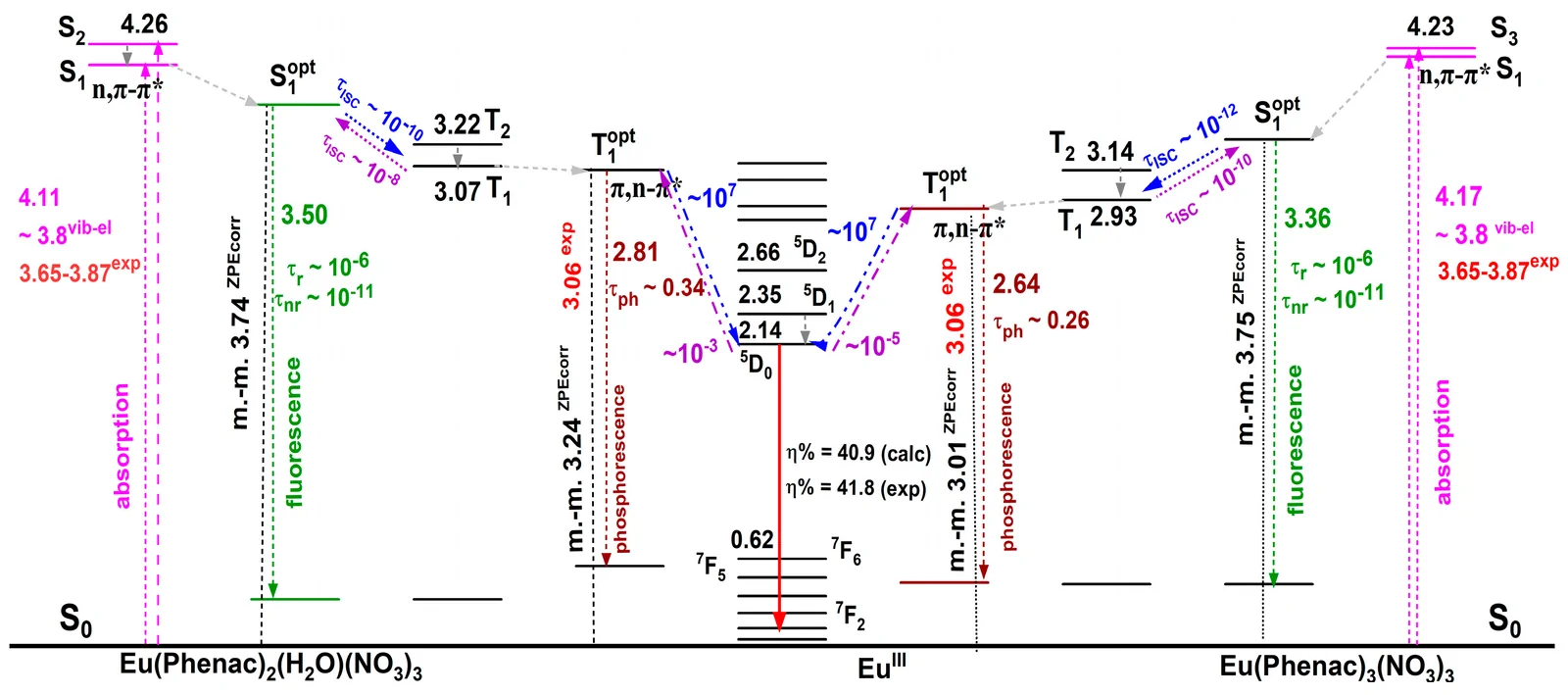
Jablonski energy level diagram calculated using the DFT/TDDFT(TDA)/ωB97XD method of Eu(Phenac)_2_(H_2_O)(NO_3_)_3_ and Eu(Phenac)_3_(NO_3_)_3_ in an acetonitrile solution. The S_1_–S_0_ fluorescence lifetimes were calculated using a procedure in MOMAP, while the most probable pathways of ISC electronic relaxation lifetimes were predicted on the basis of single-point TDDFT/RIJCOSX/ωB97XD calculations. Vertical excitation and adiabatic (ad) energies (in eV) and lifetime values (in s). The S_n_ states with the largest oscillator strengths are not drawn in the diagram.

**Table 5:** Intersystem crossing nonradiative lifetimes (τ_nr_) (S_1_ *→* T_1_ and T_1_ *→* S_1_), energy gap between donor T_1_ and acceptor ^5^D_0_ state, radiative (A_rad_) and nonradiative (A_nrad_) emission rates, energy transfer rates (W_ET_) (T_1_ *→* ^5^D_0_) and back transfer rates (italic)**,** quantum efficiency (η) and quantum yield (Φ) assessed on the basis of absorption and luminescence spectra (exp.) and of INDO/S-CIS calculations (calc.) of Eu(Phen)_2_X_3_ (X = Cl, NO_3_) incorporated into SiO_2_-based matrices [84] and Eu(Phenac)_2_(H_2_O)(NO_3_)_3_ and Eu(Phenac)_2_(NO_3_)_3_ in AN [81].

Complex	Method	τ_nr(ISC)_ [s] S_1_ → T_1_ *T_1_* → *S_1_*	ΔE [eV] (T_1_-^5^D_0_)	A_r_ [s^−1^]	A_nr_ [s^−1^]	W_ET_ [s^−1^] T_1_ → ^5^D_0_ ^5^*D_0_* → *T_1_*	η [%]	Φ [%]
Eu(Phen)_2_Cl_3_/-Si-O-Si-OH	exp			753	3457		17.86	13.53
	calc		0.47	632	3587	2.5 × 10^9^ *43.8*	14.98	14.84
Eu(Phen)_2_Cl_3_/-Si-O-Si(CH_3_)_3_	exp			457	1300		26.02	37.61
	calc		0.54	434	1324	1.8 × 10^9^ *1.5*	24.68	24.44
Eu(Phen)_2_(NO_3_)_3_/-Si-O-Si-OH	exp			365	-		-	39.60
	calc		0.66	348	529	1.2 *×* 10^9^ *1.1* × *10^−2^*	39.70	39.30
Eu(Phen)_2_(NO_3_)_3_/-Si-O-Si(CH_3_)_3_	exp			414	-		-	8.00
	calc		0.71	376	4275	1.1 × 10^9^ *1.6* × *10^−3^*	8.10	8.00
Eu(Phenac)_2_(H_2_O)(NO_3_)_3_ in AN	exp	-	(0.92)	(293)	(407)	-	(41.8)	(3.9 ± 0.40)
	calc	1.5 × 10^−10^ *1.8 × 10^−8^*	1.10	270	429	1.2 *×* 10^7^ *1.4 × 10^−3^*	38.6	38.1
Eu(Phenac)_3_(NO_3_)_3_ in AN	exp	-	0.92	293	407	-	41.8	3.9 ± 0.40
	calc	1.2 × 10^−10^ *7.2 × 10^−10^*	0.87	286	413	2.3 *×* 10^7^ *1.4 × 10^−3^*	40.9	40.4

## Data Availability

No new data were created or analyzed in this study. Data sharing is not applicable to this article.
